# Machine learning innovations in CPR: a comprehensive survey on enhanced resuscitation techniques

**DOI:** 10.1007/s10462-025-11214-w

**Published:** 2025-05-05

**Authors:** Saidul Islam, Gaith Rjoub, Hanae Elmekki, Jamal Bentahar, Witold Pedrycz, Robin Cohen

**Affiliations:** 1 Concordia Institute for Information Systems Engineering, Concordia University, Montreal, Canada; 2 Faculty of Information Technology, Aqaba University of Technology, Aqaba, Jordan; 3 Department of Computer Science, 6 G Research Center, Khalifa University, Abu Dhabi, United Arab Emirates; 4 Gina Cody School of Engineering and Computer Science, Concordia University, Montreal, Canada; 5 Department of Electrical and Computer Engineering, University of Alberta, Edmonton, Canada; 6 Systems Research Institute, Polish Academy of Sciences, Warsaw, Poland; 7 Research Center of Performance and Productivity Analysis, Istinye University, Sariyer/Istanbul, Turkey; 8 David R. Cheriton School of Computer Science, University of Waterloo, Waterloo, Canada

**Keywords:** Cardiopulmonary resuscitation (CPR), Machine learning (ML), Artificial intelligence (AI), Healthcare integration, Cardiac arrest, Reinforcement learning (RL)

## Abstract

This survey paper explores the transformative role of Machine Learning (ML) and Artificial Intelligence (AI) in Cardiopulmonary Resuscitation (CPR), marking a paradigm shift from conventional, manually driven resuscitation practices to intelligent, data-driven interventions. It examines the evolution of CPR through the lens of predictive modeling, AI-enhanced devices, and real-time decision-making tools that collectively aim to improve resuscitation outcomes and survival rates. Unlike prior surveys that either focus solely on traditional CPR methods or offer general insights into ML applications in healthcare, this work provides a novel interdisciplinary synthesis tailored specifically to the domain of CPR. It presents a comprehensive taxonomy that classifies ML techniques into four key CPR-related tasks: rhythm analysis, outcome prediction, non-invasive blood pressure and chest compression modeling, and real-time detection of pulse and Return of Spontaneous Circulation (ROSC). The paper critically evaluates emerging ML approaches-including Reinforcement Learning (RL) and transformer-based models-while also addressing real-world implementation barriers such as model interpretability, data limitations, and deployment in high-stakes clinical settings. Furthermore, it highlights the role of eXplainable AI (XAI) in fostering clinical trust and adoption. By bridging the gap between resuscitation science and advanced ML techniques, this survey establishes a structured foundation for future research and practical innovation in ML-enhanced CPR. It offers clear insights, identifies unexplored opportunities, and sets a forward-looking research agenda identifying emerging trends and practical implementation challenges aiming to improve both the reliability and effectiveness of CPR in real-world emergencies.

## Introduction

Cardiopulmonary Resuscitation (CPR) is a life-saving medical procedure that has been instrumental in the field of emergency medicine for several decades (Hurt 2005). Originating as a technique to revive individuals from drowning incidents, it has evolved into a universally recognized procedure for cardiac arrest victims, regardless of the cause (Cooper et al. 2006). At its core, CPR serves as an interim measure to simulate the heart’s function of pumping blood, ensuring that oxygenated blood continues to circulate to vital organs, especially the brain (Raza et al. 2021). This is crucial because brain cells begin to die within minutes without oxygen, leading to irreversible brain damage or death (Lee et al. 2022). With more than 400,000 cases each year in North America and an average survival rate of $10\%$, the impact of Out-of-Hospital Cardiac Arrest (OHCA) on public health is substantial (Meier et al. 2010). Despite recent technological advancements, the survival rate has been stagnating over the past few years. Management of cardiac arrest is defined by international recommendations, defined by National Resuscitation Council (Herlitz et al. 1997). It is optimized by multiple interconnected links that form the Chain of Survival. One of the most important link of this chain is CPR (Chamberlain et al. 2008). The American Heart Association and other global health organizations have emphasized the significance of CPR, not just among healthcare professionals but also among the general public (Hinkelbein et al. 2020). This is because the majority of cardiac arrests occur outside of hospital settings, where immediate medical intervention is not readily available. In such scenarios, bystander CPR can be the difference between life and death (Pellegrino et al. 2021). Despite its proven efficacy, several challenges persist in the realm of CPR. These include disparities in public awareness, variations in training methodologies, and evolving guidelines based on new research findings (Raza et al. 2021).

While traditional CPR techniques undoubtedly save countless lives and play a crucial role in the field of medical emergencies, several challenges persist. Specifically, the effectiveness of traditional CPR is hindered by the variability in human performance. Due to the dynamic nature of CPR in medical emergencies, it is very difficult for humans to consistently provide optimal pressure at the appropriate time intervals over an extended period (Ewy and Kern 2009). The recent advancements in technology and the emergence of Machine Learning (ML) offer new possibilities. To address these challenges, ML presents a potential solution to assist medical practitioners and enhance resuscitation techniques. ML algorithms can process vast amounts of data and make real-time decisions, potentially improving the accuracy and efficiency of CPR interventions. Integrating ML into CPR procedures could revolutionize the way we respond to cardiac arrests and significantly improve patient outcomes (Dahal and Ali 2022; Harford et al. 2019).

ML has emerged as a powerful tool in the medical domain, revolutionizing various healthcare applications, including CPR. The integration of ML in CPR aims to enhance survival rates by improving early detection, optimizing compression techniques, and providing real-time feedback to medical professionals. Despite significant advancements, there remain challenges in data availability, algorithm interpretability, and real-world deployment, necessitating a structured review of current ML approaches in CPR. This survey aims to provide a comprehensive analysis of ML-driven CPR innovations, evaluating existing techniques and identifying future research directions. It addresses the following key research questions:

- RQ1: What are the current applications of ML in CPR, and how do they improve patient outcomes?
- RQ2: What challenges exist in utilizing ML for CPR, particularly in terms of data availability, real-time decision-making, and model interpretability?
- RQ3: What emerging ML techniques, such as Reinforcement Learning (RL) and transformer-based architectures, hold promise for advancing CPR research?
- RQ4: How can ML models be integrated into real-world CPR practice to enhance decision-making and resuscitation effectiveness?

To answer these questions, this survey presents a structured taxonomy of ML approaches used in CPR, categorizing methods based on their applications, learning paradigms, and impact on resuscitation procedures. Additionally, we provide an in-depth discussion on RL and transformer models, highlighting their potential to address the challenges of traditional ML methods in CPR. Furthermore, we analyze the role of eXplainable Artificial Intelligence (XAI) in ensuring model transparency and clinical adoption.

The paper methodically reviews and analyzes existing ML applications in CPR, identifies research gaps, and uncovers unexplored potential, setting a new direction for empirical studies and technological advancements. This comprehensive synthesis is designed to spark innovative research and collaborations, with the goal of significantly advancing CPR techniques, ultimately impacting clinical outcomes in emergency medicine. Having noticed a gap in the existing literature that surveys exploring the integration of ML in CPR, our work aims to provide a comprehensive interdisciplinary reference. The use of CPR in emergency medicine faces several challenges due to variability in human performance, with the need to respond under pressure to problems that are dynamically changing. This paper establishes a framework for future research that demonstrates the value of ML in assisting medical practitioners and improving resuscitation techniques. The unexplored potential we uncover sets new directions for empirical studies and technological advancements. By providing this framework, we aim to contribute a valuable and novel perspective to the field.

We propose a novel taxonomy that categorizes ML approaches based on their applications in CPR tasks. This taxonomy includes four key categories:

1. Rhythm analysis: Techniques focused on accurately detecting cardiac rhythms during CPR, ensuring appropriate shock delivery.
2. Outcome prediction: Methods to predict defibrillation success and survival outcomes using advanced predictive modeling.
3. Non-invasive blood pressure and chest compression: Models for optimizing chest compression and monitoring physiological parameters.
4. Pulse and return of spontaneous circulation detection (ROSC) detection: Approaches for real-time detection of pulse and ROSC.

**Fig. 1:**
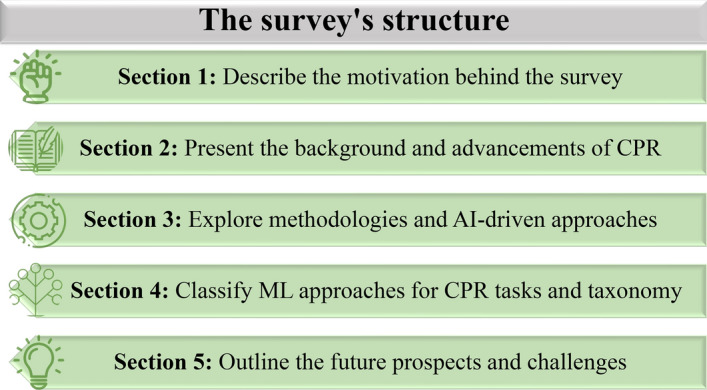
Visual abstract illustrating the structure of the survey paper. The figure outlines the key sections, including the historical background of CPR, advancements in AI-driven methodologies, classification of ML approaches, and future research directions. This structured overview provides a roadmap for readers to navigate the comprehensive discussion on the integration of ML in CPR

The novelty and primary contribution of this survey compared to previous review papers lies in its comprehensive interdisciplinary approach, systematically examining advanced machine learning methodologies specifically applied to CPR scenarios. Previous reviews typically provide broader overviews or focus narrowly on traditional resuscitation methods without integrating recent ML advancements comprehensively. In contrast, our survey explicitly categorizes, critically evaluates, and discusses recent innovations, emerging research trends, and real-world implementation challenges, thus filling a critical gap in the literature and offering clear, actionable insights to drive future research in ML-enhanced CPR techniques. The objective of this survey is to highlight the value of applying ML in the field of CPR, set against the historical background of the method, its current practices, and its potential to support new techniques. The paper is structured as follows: Sect. 2.1 provides the historical background of CPR. We present an overview of the techniques and the advancement of CPR in Sect. 3. In Sect. 4, we propose the classification of CPR studies available in the literature. Finally, in Sect. 5, we conclude the paper by summarizing our findings and discussing potential avenues for future research. Figure 1 gives a visual abstract of the whole organization of the proposed paper. The detailed abbreviations and definitions used in the paper are listed in Table 1.

**Table 1:** List of abbreviations and acronyms used in the paper

Abbreviation	Definition
AED	Automated external defibrillator
AI	Artificial intelligence
ANN	Artificial neural network
CNN	Convolutional neural network
CPR	Cardiopulmonary resuscitation
ECG	Electrocardiogram
ECPR	Extracorporeal CPR
KNN	K-Nearest Neighbors
LLM	Large language model
LSTM	Long short-term memory
ML	Machine learning
DL	Deep learning
OHCA	Out-of-hospital cardiac arrest
POCUS	Point-of-care ultrasound
RF	Random forest
REBOA	Resuscitative endovascular balloon occlusion of the aorta
RQI	Resuscitation quality improvement
RL	Reinforcement learning
DQN	Deep Q-network
HFRL	Human feedback reinforcement learning
ROSC	Return of spontaneous circulation detection
SVM	Support vector machine
XAI	Explainable artificial intelligence
VT	Ventricular tachycardia
VF	Ventricular fibrillation
PEA	Pulseless electrical activity
AS	Asystole
PR	Pulse-generating rhythm
RLS	Recursive least squares
LDB	Load-distributing band

## Background

### Historical background

The history of CPR has evolved dramatically from early airway management techniques in the 16th century to systematic approaches developed in the 18th century. Key advancements include the refinement of mouth-to-mouth resuscitation in the late 1950s and the progression of cardiac massage from open chest methods in 1874 to closed chest techniques by 1960. Significant improvements in electrical defibrillation also occurred, starting with internal use in 1947 and moving to external applications by 1956 (Cooper et al. 2006). Throughout the 1980s and 1990s, research efforts focused on refining CPR guidelines, improving defibrillation methods, and standardizing training protocols (DeBard 1980; Herlitz et al. 1997). These foundational studies played a pivotal role in shaping modern resuscitation techniques and have led to the integration of technological advancements in CPR delivery.

**Fig. 2:**
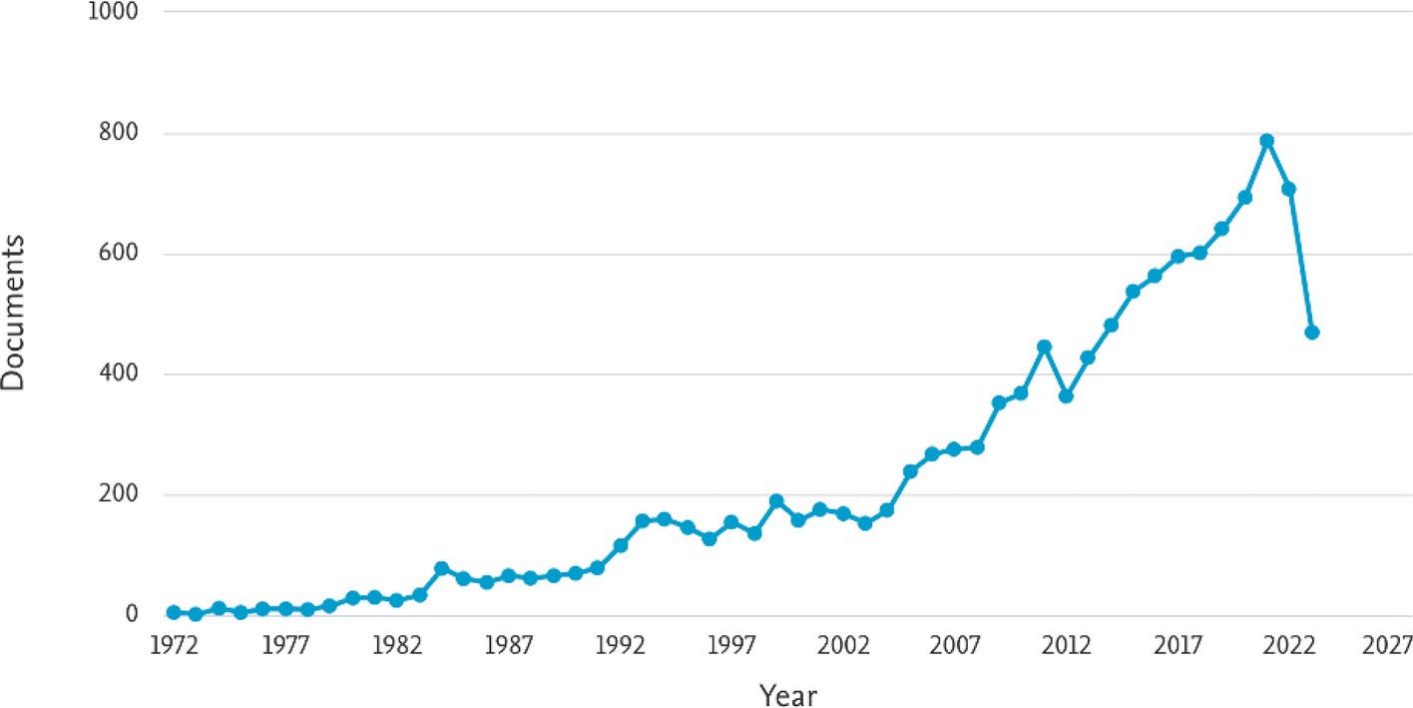
Trend of CPR-related research publications over time, generated using the Scopus Analyze Search Results tool. The figure illustrates the growing interest in CPR research, highlighting periods of increased publication activity

As research on CPR continued to evolve, the early 2000s witnessed the widespread adoption of AEDs and an increased focus on compression-only CPR, which significantly improved survival rates. These advancements laid the groundwork for the integration of AI and ML into modern resuscitation methods, enabling real-time decision support, adaptive feedback systems, and predictive modeling for patient outcomes.

**Fig. 3:**
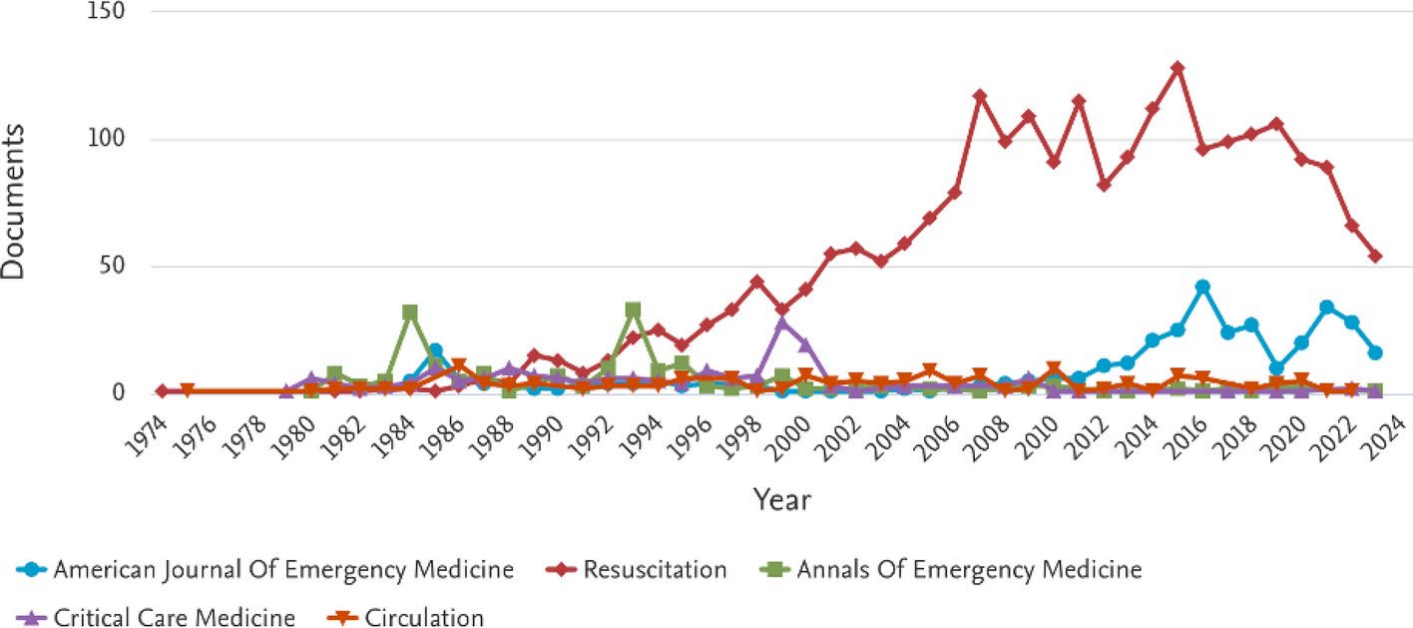
Annual distribution of CPR-related publications from various sources, generated using the Scopus Analyze Search Results tool. Each line represents a different source, showing fluctuations in research contributions over the years. Peaks indicate periods of heightened focus on CPR research, while declines may reflect shifts in research priorities or refinements in methodologies. The figure highlights how different research communities have contributed to the evolution of CPR studies

In recent years, AI and ML have emerged as transformative tools in CPR research Alowais et al. (2023); Liu et al. (2023), analyzing large-scale patient data, optimizing resuscitation protocols, and assisting healthcare providers with decision-making during cardiac arrest situations. Advances in deep learning models, real-time sensor integration, and predictive analytics are now shaping the future of CPR by improving response times and personalizing resuscitation strategies.

Today, CPR has become a globally recognized emergency procedure, continuously refined through research and technological advancements. Recent innovations in ML and AI enable real-time data analysis to enhance CPR precision, leading to improved outcomes. These advancements promise to further increase CPR’s effectiveness and save more lives. Figure 2 is a time-series line chart showing the number of CPR-related research publications over the years. The graph indicates increasing interest in CPR research, with plateaus suggesting periods of methodological refinement.

**Fig. 4:**
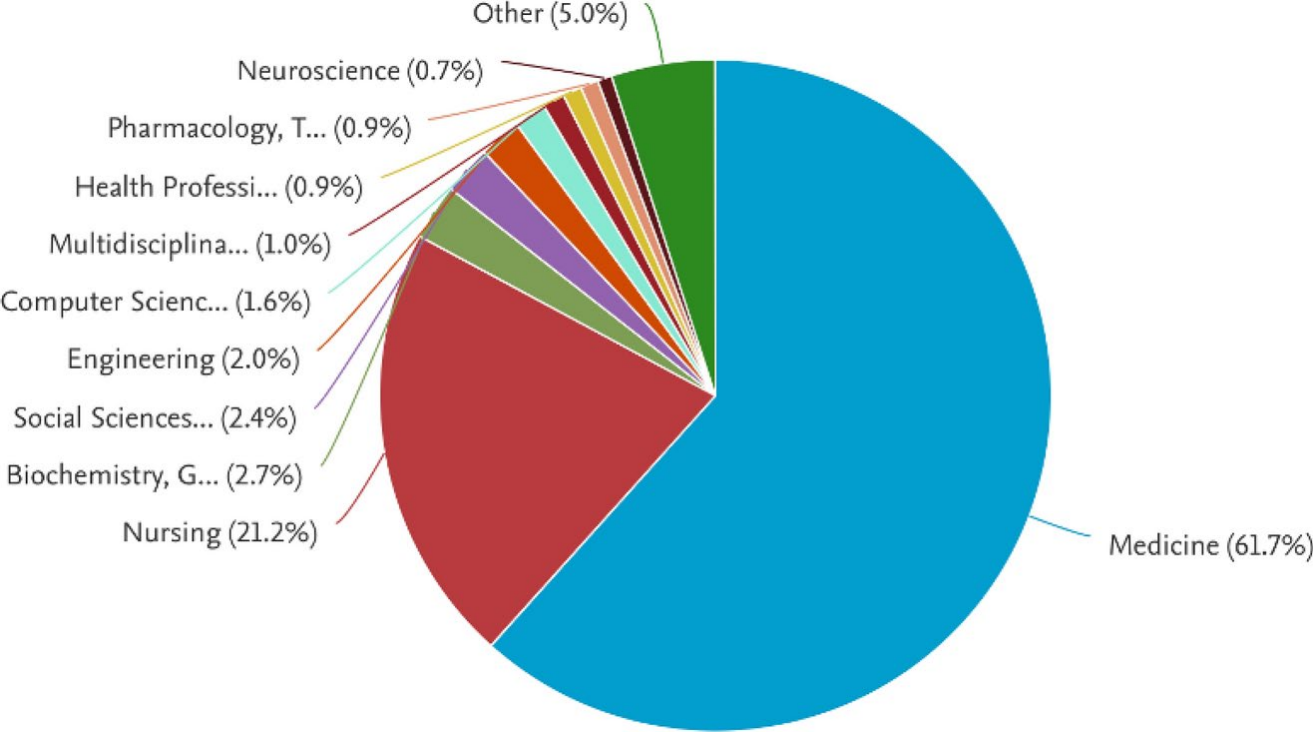
Subject-area contributions to CPR research, generated using the Scopus ’Analyze Search Results’ tool. The figure illustrates the interdisciplinary nature of CPR studies, highlighting the predominant research domains such as cardiology, emergency medicine, and computer science

Moreover, Fig. 3 illustrates how CPR research contributions have evolved over time, showing variations in research intensity and highlighting interdisciplinary efforts from diverse scientific domains. Furthermore, Fig. 4 presents the subject areas and their contributions to CPR knowledge. The figure underscores the interdisciplinary nature of CPR research, with significant contributions from cardiology, emergency medicine, and biomedical engineering, as well as emerging roles in computational sciences and AI-driven decision support.

### Historical techniques

Over time, CPR has evolved alongside advancements in medicine. By the end of the 19th and early 20th centuries, the role of oxygenation and circulation became increasingly important to the success of resuscitation efforts. While some foundational references cited in this review date back several decades, these works are intentionally included due to their critical role in shaping the foundational understanding and evolution of CPR methodologies. Tracing these early studies provides essential context for appreciating recent advancements and clearly illustrates the progressive integration of machine learning into resuscitation practices, a primary focus of our review. In fact, the most significant evolution in CPR occurred with the introduction of closed-chest cardiac massage combined with mouth-to-mouth resuscitation.

- Mouth-to-mouth resuscitation: Mouth-to-mouth resuscitation, widely recognized as a lifesaving technique in the 20th century, actually has roots that trace back to ancient practices. Studies, such as the one conducted by Poulsen et al. (1959), have demonstrated that this method can provide adequate alveolar ventilation. However, it also carries risks, including the possibility of hypoventilation. This highlights the importance of proper training in different resuscitation techniques to ensure effective emergency responses.
- Closed-chest cardiac massage: In the mid-20th century, Kouwenhoven et al. (1960) demonstrated that rhythmic chest compression could generate external blood flow. This non-invasive method, replacing manual heart massage through surgery, allows for easy, equipment-free performance, significantly improving OHCA survival rates. Despite potential cardiac injuries (Baldwin and Edwards 1976; Park 2017), the benefits outweigh the risks compared to no intervention.
- Transition to modern advancements: During the 1980s and 1990s, research focused on refining CPR techniques, improving defibrillation technology, and understanding the physiological mechanisms of resuscitation (Herlitz et al. 1997; DeBard 1980; Cummins et al. 1986). These studies laid the groundwork for automated and AI-assisted CPR interventions, leading to recent advancements in ML applications, sensor-based feedback devices, and remote assistance technologies.

### Modern advancements

The landscape of CPR has witnessed profound transformations in recent decades, with the advancements in medical science leading to improved techniques and tools for first responders and medical professionals, expanding life-saving possibilities.

- Automated external defibrillators (AEDs): All studied AEDs meet diagnostic rhythm performance standards in real OHCA scenarios, with errors typically due to operator issues rather than device faults (Zijlstra et al. 2017). Fully automatic AEDs enhance correct shock delivery, and improving emergency cardiac care outcomes.
- Compression-only CPR: Compression-only CPR maintains organ blood flow during cardiac arrest and simplifies the process by eliminating mouth-to-mouth ventilation (van Eijk et al. 2024). Studies show compressions are crucial in early cardiac arrest for sufficient blood oxygen (Sayre et al. 2008).
- Mechanical CPR devices: Mechanical CPR devices, which deliver consistent and optimal compressions, reduce the physical exertion required from rescuers. They are especially beneficial during patient transport or challenging manual compression situations (Rubertsson et al. 2014).
- Point-of-care ultrasound (POCUS): POCUS has transformed cardiac arrest management by enabling real-time heart visualization, allowing clinicians to quickly identify and treat reversible causes. It also guides interventions like fluid administration and medication adjustments based on real-time cardiac responses (Gaspari et al. 2016).
- Feedback devices in CPR: Skorning et al. (2010) describe a visual feedback device that improves chest compression performance in CPR simulations, enhancing rate, depth, and sufficiency. Its simplicity makes it suitable for trained responders, reinforcing the chain of survival and potentially improving cardiac arrest outcomes.
- Telemedicine and remote guidance: Telemedicine integrates into CPR by providing real-time guidance via video calls, notably improving chest compression rate and accuracy over audio-only instructions. However, it may slightly delay bystander CPR initiation in simulated settings (Lin et al. 2018).
- Integration of AR-VR technology: AR and VR technologies expand CPR training by enabling practice in realistic scenarios, yet wider adoption is needed in this promising research area (Semsarian et al. 2015).
- AI-driven CPR and ML integration: Recent research has explored the role of deep learning models in optimizing CPR efficiency, predicting resuscitation outcomes, and improving chest compression quality through real-time feedback systems. These advancements highlight the increasing automation and precision of modern CPR interventions (van Eijk et al. 2024; Liu et al. 2023; Islam et al. 2025).

## CPR methodologies and AI-driven approaches

### Methodologies

CPR research has evolved through a variety of methodologies that assess the effectiveness of interventions, evaluate survival outcomes, and explore emerging technologies such as ML. The studies summarized in Table 2 utilize different methodological approaches, including observational studies, literature reviews, meta-analyses, clinical trials, scoping reviews, and experimental modeling to provide insights into various aspects of CPR.

Observational studies analyze real-world CPR practices, often examining factors such as bystander intervention, compression quality, and patient survival rates. These studies provide empirical data on the effectiveness of CPR techniques under different conditions. Literature reviews aggregate findings from previous research, offering a structured synthesis of existing knowledge while identifying gaps and future research directions. Meta-analyses statistically combine results from multiple studies to assess the overall efficacy of specific CPR interventions, such as compression-only CPR or mechanical devices. Clinical trials test new CPR techniques, including mechanical compression devices and feedback systems, in controlled environments to validate their impact on survival rates and resuscitation quality.

Scoping reviews, as seen in some of the studies in Table 2, examine a broader range of literature to explore emerging trends in CPR, such as the role of AI and digital health technologies in resuscitation efforts. Additionally, experimental modeling is increasingly used to develop AI-driven CPR applications, where ML algorithms analyze Electrocardiogram (ECG) signals, predict defibrillation success, and optimize compression techniques. These computational approaches enable real-time decision-making, enhancing CPR effectiveness beyond traditional manual techniques.

Table 2 provides a comparative analysis of these methodologies, illustrating how CPR research has transitioned from conventional observational and clinical approaches to AI-driven models. This shift reflects the growing role of computational and data-driven strategies in optimizing resuscitation practices and improving patient outcomes.

**Table 2:** Detailed comparison of references for CPR advancements and challenges

References	Year	Methodology	Application	Impact
Zijlstra et al. (2017)	2017	Observational study	Public settings	Increased survival rates with AEDs
Sayre et al. (2008)	2008	Literature review	Early-stage CPR	Compression-only CPR as effective as traditional
Hunt et al. (2014)	2014	Meta-analysis	CPR training	Enhanced outcomes with high-fidelity manikins
Teeter et al. (2018)	2018	Observational study	Trauma-induced cardiac arrest	Potential benefits of Resuscitative Endovascular Balloon Occlusion of the Aorta (REBOA)
White et al. (2018)	2018	Meta-analysis	Advanced resuscitation	Improved outcomes with advanced airway management
Rubertsson et al. (2014)	2014	Clinical trial	CPR delivery	Mechanical CPR devices as effective as manual
Gaspari et al. (2016)	2016	Observational study	Cardiac arrest diagnosis	POCUS aids in determining arrest causes
Nielsen et al. (2013)	2013	Clinical trial	Post-resuscitation care	Benefits of therapeutic hypothermia
Bartos et al. (2020)	2018	Observational study	Refractory cardiac arrest	Benefits of ECPR
Skorning et al. (2010)	2015	Clinical trial	CPR training	Improved compression quality with feedback devices
Blewer et al. (2011)	2017	Observational study	Bystander CPR	Increased CPR rates with public training
Duff et al. (2018)	2018	Literature review	Pediatric CPR	Unique considerations for pediatric patients
Semsarian et al. (2015)	2016	Literature review	Sudden cardiac deaths	Technology aids in determining causes
Thakur et al. (2023)	2023	Descriptive cross-sectional study	Neonatal Intensive Care Unit	Technological advancements improve neonatal survival
Wyckoff et al. (2022)	2022	Literature review and consensus	CPR	Latest consensus on CPR and emergency care
Aldridge et al. (2022)	2022	Scoping review	Bystander CPR during emergency calls	Barriers and facilitators to bystander CPR
Ijuin et al. (2022)	2022	Observational study	ECPR in hybrid ER	Utility of hybrid ER for ECPR decisions

**Fig. 5:**
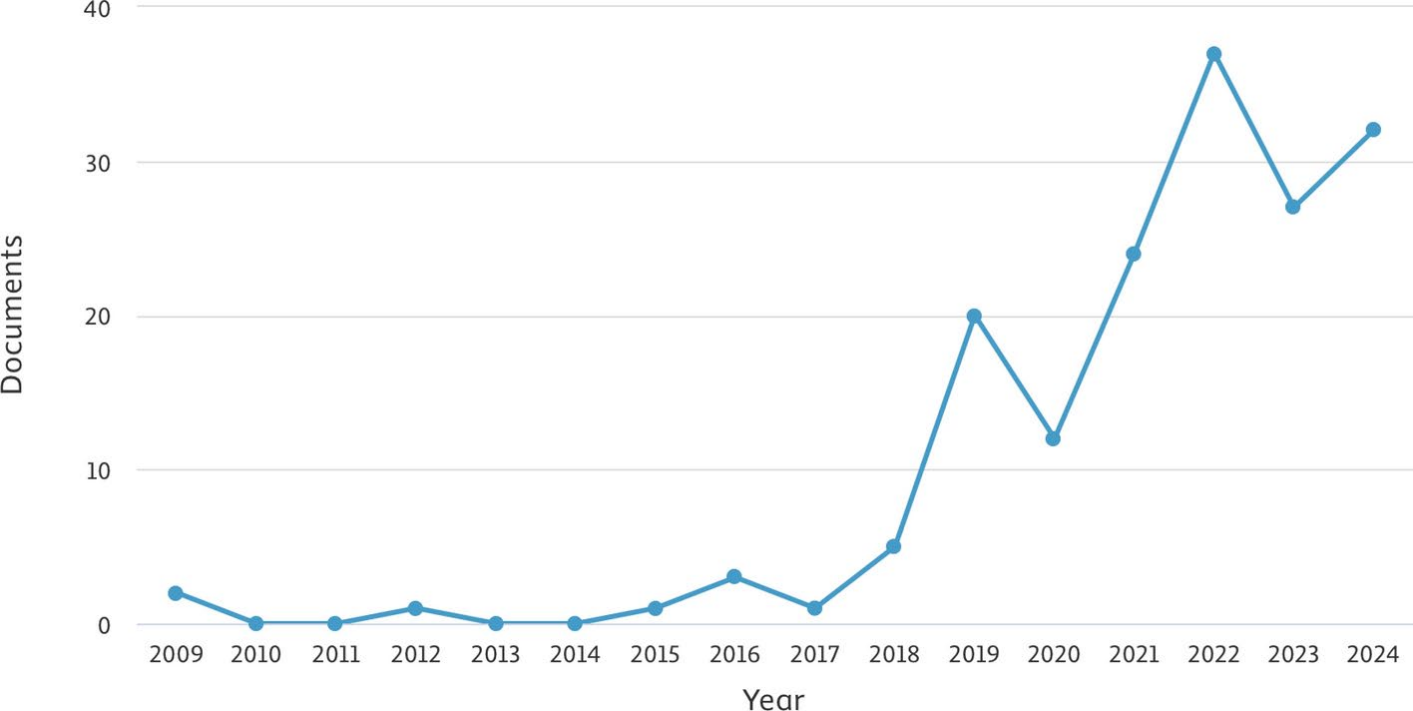
Annual trend of CPR-related publications incorporating AI and ML, generated using the Scopus Analyze Search Results tool. The figure illustrates the increasing adoption of AI and ML in CPR research over time. The rising trajectory highlights the growing interest in leveraging advanced computational techniques for improving resuscitation outcomes, while fluctuations may indicate shifts in research focus or technological advancements

The application of ML has become increasingly prominent in medicine, as it supports healthcare professionals in analyzing and diagnosing various health conditions, with a particular focus on cardiovascular diseases (Bhushan et al. 2023; Patel et al. 2022). In particular, CPR is one area where ML is beginning to play a prominent role, especially with advancements in devices and technology. These developments are aiding in the refinement and automation of CPR techniques, leading to improvements in resuscitation practices. Figure 5 illustrates the increasing adoption of AI and ML technologies in CPR research over recent years. The x-axis tracks the progression of years, while the y-axis quantifies the number of studies or documents, indicating a rising interest in leveraging these advanced algorithms for CPR enhancement. This trend reflects the benefits of AI and ML in data analysis, rhythm detection, and predictive modeling within resuscitation efforts, highlighting a promising frontier in the field.

## Classification of ML approaches for CPR tasks

### Taxonomy

**Fig. 6:**
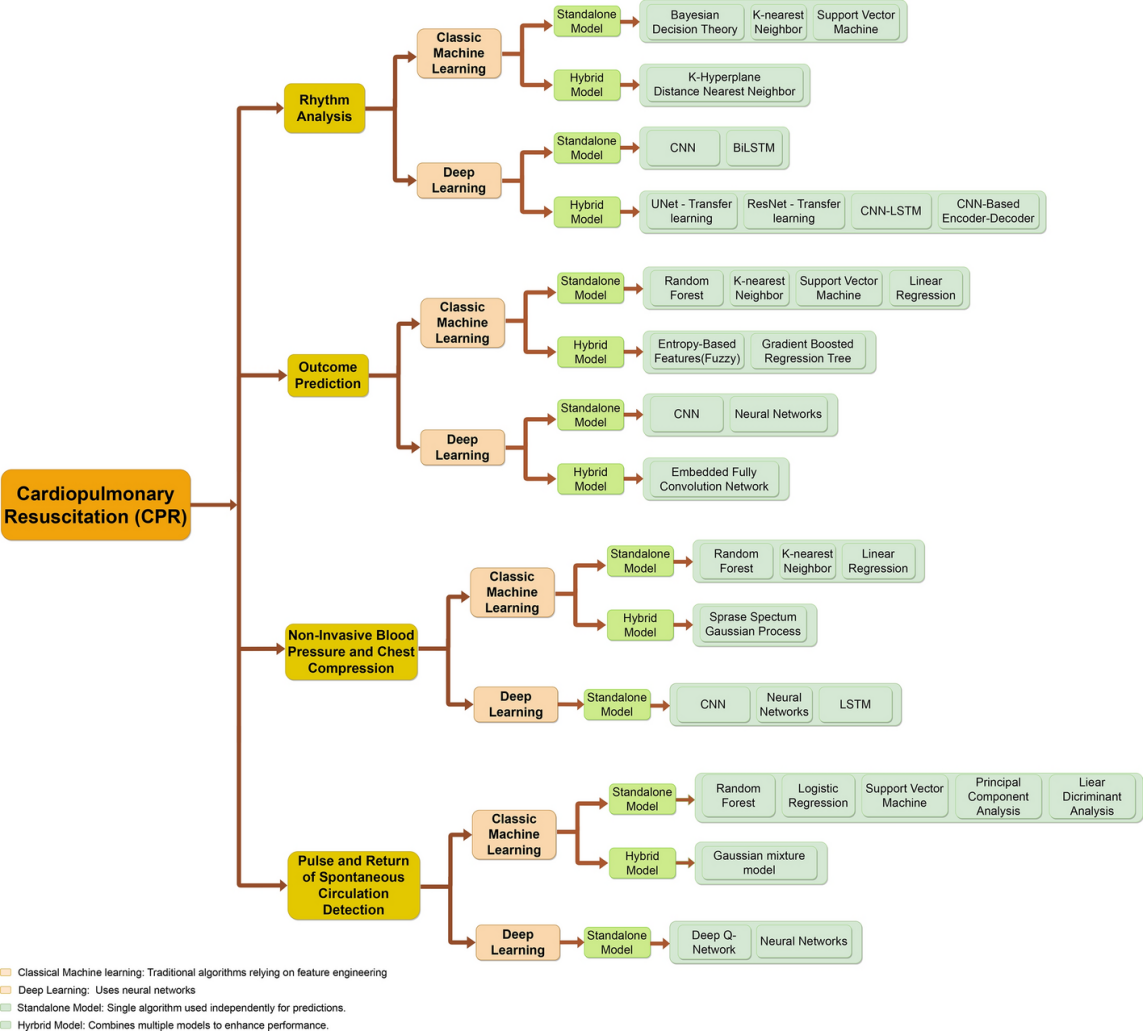
Taxonomy of ML-driven CPR tasks: a classification of ML models for four different tasks of CPR

The integration of ML and AI in CPR has led to various innovations. These advancements range from early detection of cardiac arrest during emergency calls to the optimization of chest compressions and ventilation. The upcoming part of this paper provides a comparative overview of several studies that have contributed to these machine-driven innovations in CPR. We present the ML techniques and classes covered in each publication along with the details of the application fields for each model, and their key contributions, and provide a concise summary of the paper. We conducted thorough research across different databases: Pubmed, Google Scholar, IEEE, Science Direct, and ACM to find papers introducing new ML-based models aimed at CPR. The findings were classified into four categories according to CPR tasks: (i) Rhythm Analysis; (ii) Outcome Prediction; (iii) Non-Invasive Blood Pressure and Chest Compression; and (iv) Pulse and ROSC. In Fig. 6, we introduce a taxonomy for ML approaches used in CPR tasks, where we focus on the top four tasks and categorized the models into two main sections: ML and Deep Learning (DL). In the ML category, we included basic and classic models, while in the DL category, we included models that use neural networks. We further divided the models into two types: standalone models and hybrid models. Standalone models use the core architecture without any significant changes, while hybrid models modify the core architecture. We identified several approaches that used standalone models for CPR tasks, as well as approaches that used hybrid models in both ML and DL.

The introduced taxonomy visualizes and provides a quick grasp of the existing ML approaches for different CPR tasks, research gaps, and possible ML/DL approaches that can be experimented with for these tasks. We analyze all these approaches in detail and discuss additional ML techniques for CPR tasks in this section.

### Rhythm analysis

Rhythm analysis refers to the process of identifying heart rhythms from ECG signals during CPR. These rhythms, often categorized as resuscitation cardiac rhythms, include Ventricular Tachycardia (VT), Ventricular Fibrillation (VF), Pulseless Electrical Activity (PEA), Asystole (AS), and Pulse-generating Rhythm (PR). The primary distinction between these rhythms lies in whether they are shockable (requiring defibrillation shocks), such as VF and VT or non-shockable (not requiring defibrillation shocks), such as PEA, AS, and PR. However, during CPR, chest compressions generate artifacts in the ECG signal, making it challenging to accurately differentiate between shockable and non-shockable rhythms. As a result, rhythm analysis typically necessitates an interruption of CPR to obtain a clear assessment. This section explores how ML techniques are applied to address the challenge of rhythm analysis during CPR, either with brief interruptions or without the need to stop chest compressions altogether. Table 3 depicts the existing ML/DL approaches for rhythm analysis, highlighting the techniques employed in each study along with key contributions and metrics. Below, we will review each study outlined in the table.

**Fig. 7:**
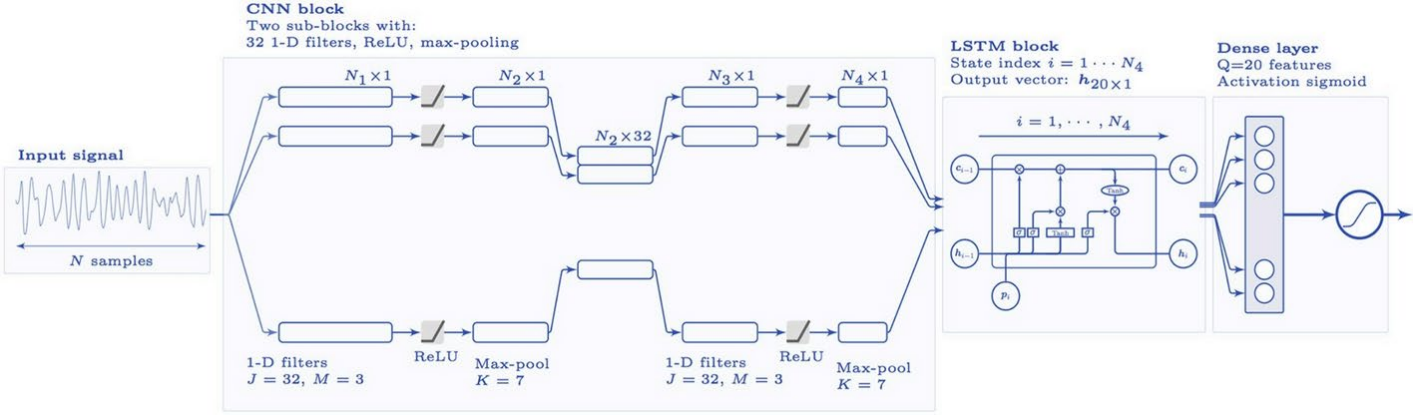
Architecture of the DL network. The architecture has three blocks: a CNN, a LSTM, and a final decision stage based on a neural network (Picon et al. 2019)

The first study reviewed is by Rad et al. (2017), which applied various ML algorithms to automatically classify the cardiac rhythms from ECG signals. However, their approach struggles with distinguishing between certain rhythms, like PR and PEA. The authors highlight the need to include additional signals beyond ECG to improve classification accuracy. Performance can be further improved using deep neural networks, as demonstrated in (Picon et al. 2019), where a classifier combining Convolutional Neural Network (CNN) and Long Short-Term Memory (LSTM), as shown in Fig. 7, is used to detect lethal ventricular arrhythmia, which is critical for guiding AED shock decisions. The inclusion of LSTM improves detection accuracy by capturing long-term temporal relationships in ECG signals. However, a major limitation of these studies is that the datasets used only contain ECG signals without CPR artifacts, which are important to address.

**Fig. 8:**
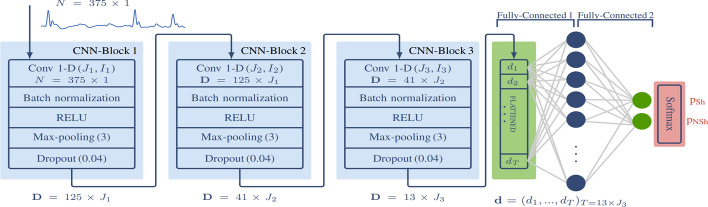
Architecture of the CNN-based shock/no-shock algorithm (Isasi et al. 2020)

In the existing literature, various filters have been proposed to remove CPR artifacts from ECG signals. For load-distributing band (LDB) mechanical chest compression devices, Isasi et al. (2021) use the recursive least squares (RLS) filter with reference signals like thorax impedance to eliminate artifacts and adapt to the dynamic nature of chest compressions. ML models such as SVM and RF are then used for shock decision-making. In this study, SVM performs well due to its capacity to handle complex, non-linear data, such as ECG signals. However, SVM struggles with large datasets, highlighting the need for deep neural networks. Another approach, using CNNs, is presented by Isasi et al. (2020), where a RLS filter is applied to remove CPR-induced artifacts before performing rhythm classification, as illustrated in Fig. 8. Nevertheless, these methods often rely on additional reference signals, such as thorax impedance, compression depth, and chest acceleration, which are not available in most commercial external defibrillators (Hajeb-M et al. 2022).

Alternatively, some methods analyze rhythms directly without pre-filtering artifacts. For instance, Hajeb-M et al. (2021) propose a CNN-based approach to classify shockable and non-shockable rhythms, both with and without CPR artifacts. In their method, the 1D ECG signal is transformed into 2D images by combining amplitude and phase components, enabling the CNN to learn spatial features. They also incorporate a BiLSTM network to capture long-term temporal dependencies in the signal. Similarly, Jekova and Krasteva (2021) present a CNN-based solution for determining whether a shock should be delivered in the presence of corrupted ECG signals. They evaluate different CNN models to identify the most effective one based on the highest balanced accuracy (BAC), using a random search approach.

Interrupting chest compressions for rhythm classification and using short ECG segments without artifacts is another approach, as demonstrated by Jaureguibeitia et al. (2020). They propose an DL-based shock decision system that uses CNN and ResNet to classify cardiac rhythms using ECG segments. This method briefly pauses chest compressions for rhythm analysis without affecting survival chances. However, short ECG segments may not provide enough information for accurate rhythm classification, which is why longer signals are often preferred for better performance.

ML presents a promising alternative, enabling both the filtering and the classification. A significant contribution in this field is the work by Gong et al. (2023), who introduced an ML-based approach to restore the ECG signals during chest compressions and classify rhythms using clean ECG waveform. Their approach combines a self-supervised UNet to clean the ECG signal and improve its signal-to-noise ratio (SNR), along with a ResNet for rhythm classification. The model also incorporates transfer learning and uses a simulated dataset with ECG signals corrupted by random SNR variations. The approach shows good performance even when evaluated on a testing dataset that is independent of the training dataset. Similarly, Hajeb-M et al. (2022) employed a CNN-based encoder-decoder model to remove artifacts from ECG signals, thereby restoring clean rhythm data for shock/no-shock classification. In their study, the authors generated a synthetic dataset of CPR-contaminated shockable and non-shockable ECG signals for training and evaluation of the proposed model.

**Table 3:** Comparative overview of ML approaches for rhythm analysis tasks of CPR

References	AI/ML techniques	Key contribution	Metrics
Gong et al. (2023)	UNet, ResNet, Transfer Learning	ECG signal restoration for accurate shock decision-making	BAC, Spe, Sen, Accuracy (Acc)
Hajeb-M et al. (2021)	CNN, BiLSTM	ECG signal classification & without CPR artifact removal	Sen, Spe, Acc, F1-Score
Isasi et al. (2020)	CNN	ECG signal filtering & classification	BAC, Spe, Sen, Acc, NPV, PPV
Jaureguibeitia et al. (2020)	CNN, ResNet	Short ECG signal classification without prior CPR artifact removal	Spe, Sen, Acc, BAC
Rad et al. (2017)	Bayesian Decision Theory, K-Nearest Neighbors (KNN), ANN, Decision Tree	Automatic classification of the cardiac rhythms	Sen, PPV
Picon et al. (2019)	CNN-LSTM	Detection of shockable arrhythmia during OHCA	BAC, Spe, Sen, Acc, NPV, PPV, AUC
Jekova and Krasteva (2021)	CNN	Shock advisory during CPR using ECG signals without filtering	BAC, Spe, Sen, Acc
Isasi et al. (2021)	SVM, Logistic Regression, RF	Filtering LDB artifacts from ECG signals and classification for shock decision-making	BAC, Spe, Sen, Acc
Hajeb-M et al. (2022)	CNN-based Encoder-Decoder	Removal of CPR artifacts from ECG signals and shock/no-shock classification	Spe, Sen, Correlation Coefficient

#### Dataset

The data used in rhythm analysis studies primarily consists of ECG signals, either with or without CPR artifacts. As presented in Table 4, the majority of the studies rely on private datasets, while another group of studies make use of publicly available datasets. The commonly used public datasets include the Creighton University Tachyarrhythmia Database (CUDB) (Nolle et al. 1986), the Massachusetts Institute of Technology-Beth Israel Hospital Malignant Ventricular Arrhythmia Database (VFDB) (Greenwald 1986), the Sudden Cardiac Death Holter Database (SDDB) (Greenwald 1986), and the MIT-BIH Atrial Fibrillation Database (AFDB) (Goldberger et al. 2000). The table also provides insight into the duration of the ECG segments used in the surveyed studies. Longer segments contain more information, but their real-time processing is more demanding and may disrupt the CPR process if prolonged for too long. Moreover, it is important to note that none of the existing studies involve clinical, real-world testing. All of the works evaluate their solutions on offline-collected data, with no direct testing in live clinical environments. This limits the applicability of the results to actual emergency scenarios where real-time processing is crucial.

**Table 4:** Highlights about the datasets used for rythm analysis

References	Data type	Data organization	Data availability
Gong et al. (2023)	ECG signals, CPR Artifacts	ECG segments of 5 s. CPR artifacts simulated and combined with clean ECG signals	Public, private datasets
Hajeb-M et al. (2021)	ECG signals, CPR Artifacts	8 s segments of CPR-corrupted ECG signals	Public, private datasets
Isasi et al. (2020)	ECG, thoracic impedance and compression depth signals	ECG segments of 9 s	Private dataset
Jaureguibeitia et al. (2020)	ECG, thorax impedance signals	ECG segments of 1 s to 4 s	Private dataset
Rad et al. (2017)	ECG, thorax impedance, compression	ECG segments of 3 s including single rhythm type and without artifacts	Private dataset
Picon et al. (2019)	ECG signals	ECG segments of 2, 3, 4, 8, 10 s	Public, private datasets
Jekova and Krasteva (2021)	Clean & corrupted ECG signals	ECG segments of 10 s	Private dataset
Isasi et al. (2021)	ECG signals	ECG segments of 16 s	Public, private datasets
Hajeb-M et al. (2022)	ECG signals	ECG segments of 14 s	Public, private datasets

#### Analytical discussion

The reviewed literature highlights the significant challenge posed by CPR-induced artifacts in rhythm analysis and the classification of cardiac rhythms from ECG signals. Various approaches have been proposed, each with different strategies for addressing this issue while aiming to meet the American Heart Association’s (AHA) requirements of 95% specificity (Spe) and 90% sensitivity (Sen). Rad et al. (2017), focused on rhythm classification without artifact removal, achieving an accuracy of 78.5%, with Sen ranging from 65.91% for PR to 88.66% for AS. (Picon et al. 2019) demonstrated the effectiveness of combining CNN and LSTM for rhythm classification, achieving Sen of 99.2% sensitivity and Spe of 96.7%. Other studies, like Hajeb-M et al. (2021) and Jekova and Krasteva (2021), also classified rhythms without filtering, but with varying performance: Sen of 94.21% and Spe of 86.14% for Hajeb-M et al. (2021), and low Sen of 74.2% and Spe of 84.6% for Jekova and Krasteva (2021). In contrast, Isasi et al. (2021) and Isasi et al. (2020) used filters such as RLS combined with SVM and CNN, respectively, achieving Sen of 92.1% and 95.8%, and Spe of 96.8% and 96.1%. Another approach, as demonstrated by Jaureguibeitia et al. (2020), used short ECG segments to avoid CPR artifacts, achieving the AHA’s requirements. Finally, methods that combined filtering and classification using ML/DL techniques, such as Gong et al. (2023), and Hajeb-M et al. (2022), achieved Sen and Spe values of 91.2% and 90.6%, and 87.18% and 97.63%, respectively. Although the results of these latter methods are not the best, they offer promising solutions for real-time rhythm classification in the presence of CPR artifacts, as they clean and classify ECG signals without interrupting CPR or requiring additional signals.

### Outcome prediction

Outcome prediction in CPR involves analyzing the ECG signal characteristics, particularly before and after delivering CPR, to estimate the probability of successful defibrillation, survival, and neurological outcomes. Measures like the cardioversion outcome prediction (COP) quantify the likelihood of successful defibrillation by assessing ECG signal changes induced by CPR. Clinical prediction models using factors like initial presentation, interventions, and time intervals can also reliably estimate survival probabilities in OHCA patients (Box et al. 2008). Accurate outcome prediction can guide appropriate interventions and management decisions during resuscitation efforts.

Table 5 analyzes the research paper while different ML techniques are being applied for outcome prediction tasks in CPR. Chen et al. (2022) proposed a method to predict invasive coronary perfusion pressure using noninvasive ECG and photoplethysmography based on ML. Harford et al. (2019) designed a ML-centered model to forecast survival after cardiac arrest by sorting OHCA patients into two groups: those experiencing positive neurological outcomes and those encountering adverse neurological outcomes or mortality. Howe et al. (2014) utilized VF waveform metrics and SVM for predicting defibrillation success, enhancing CPR by optimizing the timing of defibrillation. Entropy-based features were employed by another study (Chicote et al. 2016) to predict defibrillation success in cardiac arrest, improving shock outcome prediction accuracy. He et al. (2016) combined amplitude spectrum area with previous shock information using neural networks, enhancing the predictability of defibrillation outcomes in OHCA scenarios. In a similar advancement, Shandilya et al. (2016) developed an MDI model that incorporates non-linear dynamics for robust prediction of defibrillation success, outperforming traditional methods. In this context, the work of Coult et al. (2019) represents a significant advancement. They developed an algorithm capable of predicting defibrillation outcomes during chest compressions, achieving an Area Under the Receiver Operating Characteristic (AUROC) of 0.74 with chest compression artifacts, compared to 0.77 without. This high level of calibration suggests that the algorithm could serve as a reliable probability index for defibrillation outcome predictions. As of now, no studies have been published on the use of DL for this purpose, but the recent work by Ivanović et al. (2020) using CNN for ECG signal analysis indicates promising directions. Additionally, the integration of logistic regression and SVM by Coult et al. (2021) to analyze ECG data during CPR without needing to stop compressions, combining predictive modeling for both short-term and long-term outcomes, showcases the continuous evolution of ML applications in improving CPR effectiveness.

ML algorithms have revolutionized outcome prediction in CPR by enabling real-time analysis of ECG signals and patient physiological parameters. These models provide accurate predictions for defibrillation success, survival probabilities, and neurological outcomes, aiding healthcare professionals in making data-driven decisions during critical moments. Through predictive modeling, ML enhances the precision of intervention strategies, optimizes patient outcomes, and supports effective resuscitation protocols. Table 5 summarizes various ML approaches applied to outcome prediction tasks, illustrating their methodologies, metrics, and key contributions. These techniques showcase the growing integration of ML in improving CPR outcomes and guiding clinical decision-making processes.

**Table 5:** Comparative overview of ML approaches for outcome prediction tasks of CPR

References	AI/ML techniques	ML class	Key contribution	Metrics
Coult et al. (2019)	SVM	Supervised learning	Evaluated the predictive performance of VF waveform measures during ongoing chest compressions	Sen, Spe, OC curve
Harford et al. (2019)	Embedded Fully Convolutional Network	Supervised learning	Prediction of neurological outcomes of patients	Sen
Chen et al. (2022)	Support vector regression, RF, KNN, Gradient boosted regression tree	Supervised Learning	Prediction of invasive coronary perfusion pressure (CPP)	Correlation Coefficient, Mean Absolute Error (MAE), Root Mean Square Error (RMSE), Adjusted Determination Coefficient (R^2^)
Shandilya et al. (2016)	Multiple Domain Integrative Model, Non-linear Dynamics	Supervised Learning	Developed an MDI model for robust prediction of defibrillation success	AUC, Sen, Sep, Acc
He et al. (2016)	Neural Networks	Supervised Learning	Improved prediction performance of defibrillation outcome	AUC, Negative Predictive Value (NPV), Sen, Sep, Prediction Accuracy (PA)
Chicote et al. (2016)	Entropy-based Features (Fuzzy Entropy)	Supervised Learning	Predict defibrillation success in cardiac arrest, improving shock outcome prediction accuracy	Sen, Sep, AUC
Howe et al. (2014)	SVM	Supervised Learning	Utilized VF waveform metrics and predicting defibrillation success, enhancing CPR by optimizing the timing of defibrillation	Acc, Sen, Sep, AUC
Ivanović et al. (2020)	CNN	Supervised Learning	Predicting defibrillation success by analyzing ECG signals, achieving high accuracy and implementation	Sen, Sep, NPV, Acc
He et al. (2016)	Neural Networks	Supervised Learning	Improved prediction performance of defibrillation outcome	AUC, Sen, Sep, NPV, PPV
Coult et al. (2021)	LR/SVM	Supervised Learning	Developed an algorithm to predict outcomes of VF shock during CPR, enhancing decision-making in real-time	AUC

#### Dataset

Most of the cited studies utilized private datasets for model training and evaluation. However, a few exceptions provided access to their datasets or described their data characteristics in detail (see Table 6). For instance, He et al. (2016) provided a spreadsheet containing data collected from the emergency departments of Southwest Hospital and Xinqiao Hospital in Chongqing between January 2012 and February 2014. Similarly, Shandilya et al. (2016) relied on data provided by the Richmond Ambulance Authority (Richmond, VA) and Zoll Medical Corp. (Chelmsford, MA). Additionally, Ivanović et al. (2020) utilized publicly available ECG datasets, which facilitated reproducibility and further research. These datasets often consist of ECG signals, patient demographics, and other physiological parameters critical for predicting CPR outcomes. While these resources have been invaluable, the broader field still faces a significant challenge: the limited availability of large-scale, high-quality, and diverse datasets for developing and validating ML models in outcome prediction tasks.

**Table 6:** Highlights about the datasets used for outcome prediction

References	Data type	Data organization	Data availability
Coult et al. (2019)	VF waveform	Waveform segments analyzed with ongoing chest compressions	Private dataset
Harford et al. (2019)	Neurological outcome data	Patient records categorized by neurological outcomes	Private dataset
Chen et al. (2022)	ECG, PPG signals	ECG and PPG signals used to predict CPP	Public, private datasets
Shandilya et al. (2016)	ECG signal	Multiple-domain ECG features extracted	Private dataset (Richmond Ambulance Authority, Zoll Medical Corp.)
He et al. (2016)	ECG waveform	Amplitude spectrum and shock information integrated	Public dataset (Southwest Hospital, Xinqiao Hospital)
Chicote et al. (2016)	ECG entropy-based features	Entropy-based ECG features extracted for defibrillation success	Public dataset
Howe et al. (2014)	VF waveform metrics	VF waveform analyzed with ML	Private dataset
Ivanović et al. (2020)	ECG waveform	CNN applied on raw ECG signals for feature learning	Public dataset
Coult et al. (2021)	VF shock data	Logistic Regression and SVM applied on VF shock data	Private dataset

#### Analytical discussion

The reviewed studies emphasize the transformative role of ML in CPR outcome prediction, particularly for defibrillation success. He et al. (2016) showcased the potential of neural networks by integrating amplitude spectrum area (AMSA) with prior shock information (PSI), achieving an AUC of 0.904. Similarly, Chicote et al. (2016) utilized entropy-based features, achieving a sensitivity of $80.4\%$ and specificity of $76.9\%$.

Building on this, Ivanović et al. (2020) introduced a novel DL approach using CNNs to predict defibrillation outcomes from raw VF waveforms. Unlike traditional methods relying on hand-crafted features, this model automatically learned optimal features through its architecture. The CNN achieved an accuracy of $93.6\%$, sensitivity of $98.8\%$, and specificity of $88.2\%$, outperforming traditional classifiers like RF $(82.8\%)$ and SVM $(81.5\%)$. These results highlight the superiority of automated feature extraction over conventional ML methods. Additionally, the dataset used in this study was made publicly available, promoting replicability and further research in this domain. Shandilya et al. (2016) further validated the robustness of SVM models for VF waveform analysis, achieving an AUROC of 0.75 during chest compressions. Moreover, Howe et al. (2014) leveraged a Gaussian radial-basis-function SVM to combine VF waveform features, achieving a predictive accuracy of 81.9% and sensitivity of 87.6%.

Despite these advances, challenges persist in accessing large, diverse datasets, as most studies rely on proprietary data. Future research should explore the integration of advanced architectures like Transformers for sequential data processing and Reinforcement Learning (RL) for patient-specific adaptive interventions. This analysis underscores the significant strides made by ML models, particularly CNNs and SVMs, in enhancing CPR-related outcomes. Prioritizing the development of open-access datasets and refining advanced algorithms will be essential for driving further advancements in real-time decision-making during resuscitation.

### Non-invasive blood pressure and chest compression

Non-invasive blood pressure is a method to measure blood pressure without needing to insert anything into the body. It uses external devices like an inflatable cuff around the arm. During CPR, monitoring non-invasive blood pressure helps track the patient’s blood pressure to make sure chest compressions and other treatments are working (Hansen and Bülow 2014). On the other hand, Chest compressions are a key part of CPR. They involve pressing down on the patient’s chest to manually pump blood through the heart and circulate it around the body. Proper chest compressions are crucial to keep blood flowing to vital organs until the heart can start beating effectively on its own again (Nishiyama et al. 2010). So, non-invasive blood pressure and chest compression are co-related and crucial tasks of CPR. ML has the potential to play a transformative role in improving non-invasive blood pressure monitoring and chest compressions during CPR by enabling real-time analysis, feedback, and optimization. ML algorithms can analyze time-series non-invasive blood pressure monitoring data to identify trends and ensure adequate blood pressure is maintained. ML can provide real-time feedback on compression quality (depth, rate, recoil) and offers dynamic adjustments to improve performance. ML also predicts outcomes and detects anomalies in non-invasive blood pressure or compression parameters through unsupervised learning methods.

Table 7 summarizes relevant ML approaches for non-invasive blood pressure and chest compression tasks. While, regarding ML techniques, ML class, key contribution, and Metrics have been discussed for each of the approaches. For instance, Zhao et al. (2021) utilizes a CNN to examine chest compression depth data, distinguishing between abnormal and normal compressions to ensure CPR effectiveness. Meanwhile, Jalali et al. (2014) employs unsupervised ML to identify the optimal parameters describing chest mechanical properties during CPR. This effort aims to comprehend the intricate nature of CPR, ultimately enhancing its performance. On the other hand, Lampe et al. (2019) harnesses the power of RF algorithms to develop a predictive model that estimates carotid blood flow from chest compression parameters. Their work not only predicts but validates these estimations, paving the way for more tailored CPR techniques. Additionally, Sebastian et al. (2020) apply linear regression within a closed-loop machine-controlled CPR system that dynamically adjusts compression characteristics based on real-time hemodynamic feedback. This innovative approach optimizes coronary perfusion pressure during prolonged CPR sessions, showcasing the potential of ML to improve long-term CPR effectiveness. Similarly, a diverse array of algorithms including LSTM, neural networks, linear regression, and sparse spectrum Gaussian process are used by Gandhi et al. (2018) to demonstrate state-of-the-art modeling performance in predicting coronary perfusion pressure during CPR. This study analyzes the performance of each algorithm for single-step and long-term predictions, contributing significantly to our understanding of dynamic physiological responses during cardiac emergencies. Additionally, another study by Park et al. (2019) has designed a system that utilizes LSTM for carrying out earlobe photoplethysmography, aiming at the non-invasive estimation of blood pressure. This innovative approach holds the potential to enhance patient monitoring in real-time during CPR.

**Table 7:** Comparative overview of ML approaches for non-invensive blood pressure and chest compression tasks of CPR

References	AI/ML techniques	ML Class	Key contribution	Metrics
Gandhi et al. (2018)	LSTM, Neural Network, Linear Regression, Sparse Spectrum Gaussian Process	Supervised Learning	Analyze the performance of each algorithm for single-step and long-term predictions	RMSE
Jalali et al. (2014)	K-NN	Unsupervised Learning	Modeling mechanical properties of the chest	RMSE
Sebastian et al. (2020)	Linear Regression	Supervised Learning	Optimizing coronary perfusion pressure during CPR	Analysis of variance(ANOVA), Area under the curve(AUC)
Lampe et al. (2019)	RF	Supervised Learning	Developed a model to predict carotid blood flow from chest compression parameters	RMSE
Park et al. (2019)	LSTM	Supervised Learning	Designed a system for estimation of blood pressure	Pearson’s correlation, t-test, RMSE
Zhao et al. (2021)	CNN	Supervised Learning	Recognizing abnormal chest compression depth	Acc, F-Score

#### Dataset

Most studies on non-invasive blood pressure and chest compression tasks during CPR have relied on proprietary datasets, limiting access and reproducibility. A few datasets include human cardiac arrest cases provided by medical institutions (Zhao et al. 2021), while others have been generated using animal models. For example, (Gandhi et al. 2018), (Sebastian et al. 2020), and (Park et al. 2019) conducted experiments using pigs, and (Jalali et al. 2014) utilized 15 healthy 3-month-old female domestic swine for data collection. These studies employed diverse data types tailored to their experimental goals. Coronary Perfusion Pressure (CPP) signals were central to the work of (Gandhi et al. 2018) and (Sebastian et al. 2020), whereas chest compression data was prominently used by (Jalali et al. 2014) and (Zhao et al. 2021). Table 8 summarizes the data organization and methodologies applied in these non-invasive blood pressure and chest compression tasks during CPR.

**Table 8:** Highlights about the datasets used for non-invasive blood pressure and chest compression

References	Data type	Data source	Data organization
Gandhi et al. (2018)	Coronary Perfusion Pressure Signal	An adult female pig	Sampling rate of 25 Hz using piezoelectric pressure sensor
Jalali et al. (2014)	Force-compression data	Animal- swine	10273 CPR cycle used for the experiment (shorter than 0.4s and longer than 1 s have been excluded)
Sebastian et al. (2020)	Coronary Perfusion Pressure Signal	24 naïve farm-raised female pigs	Maximum of 6 cm for compression amplitude and 7.0 cm for decompression amplitude, with a 50% compression-decompression duty cycle
Lampe et al. (2019)	Physiological data(carotid blood flow signal)	Two Animals	Chest compressions rates of 50, 75, 100, 125, and 150 compressions per minute (cpm), depth of 5–6 cms
Park et al. (2019)	Earlobe photoplethysmography (PPG) signals	Simulated data - Pigs	CPR performed with sequence rest (10 s) - CPR (40 s) - rest (10 s) and 255 datasets generated, 135 datasets training set(VF/Asys: 87/48) and 80 datasets test set(VF/Asys: 58/32)
Zhao et al. (2021)	Chest Compression(CC) signals	Simulated data - human	Collected on ECS, 680 records as the training set and 120 records as the testing set (error of ±1 mm)

#### Analytical discussion

**Fig. 9:**
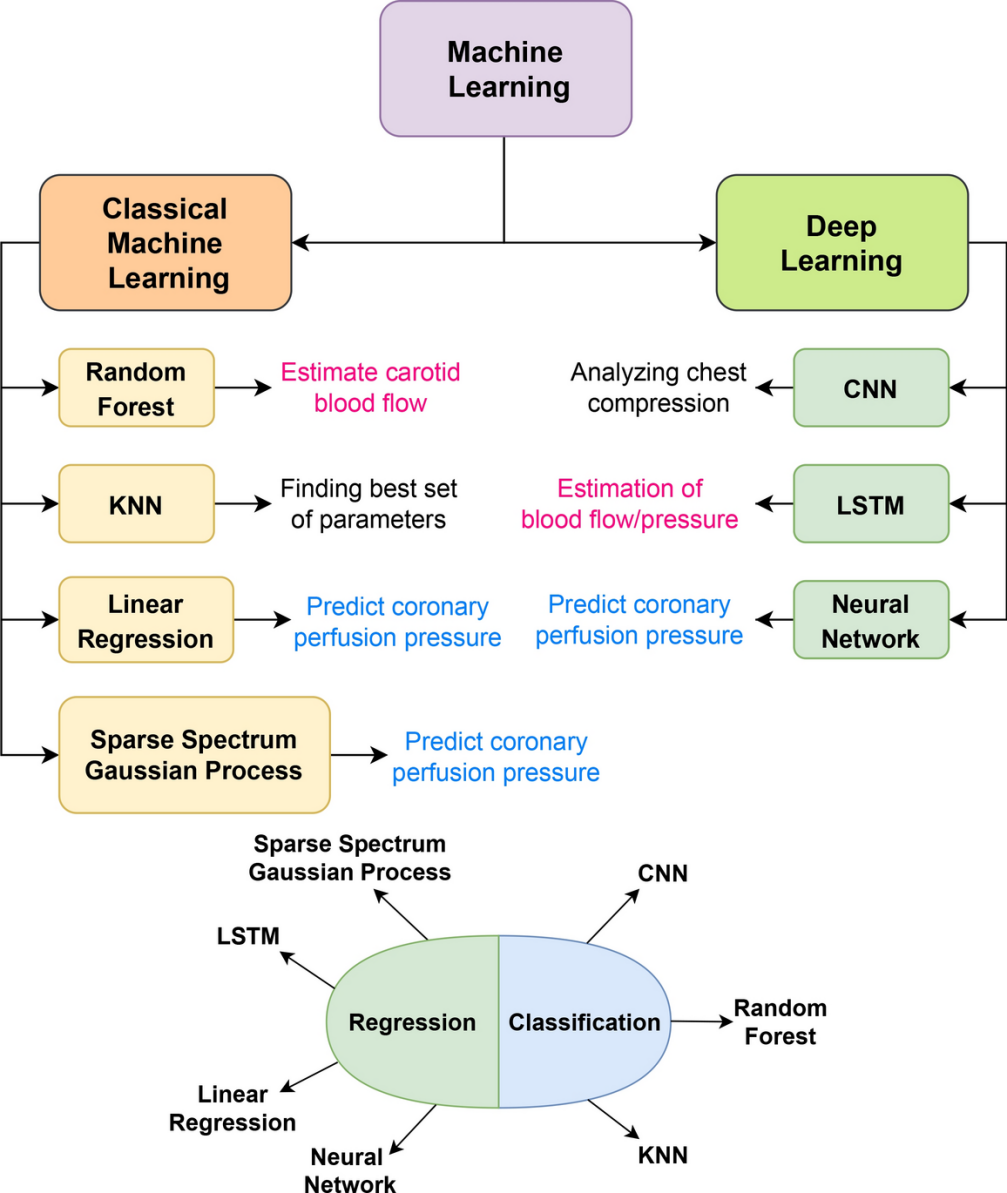
Analysis of ML models for non-invasive blood pressure and chest compression

For the Non-Invasive Blood Pressure and Chest Compression tasks in CPR, both classical ML models and neural network-based models have been utilized, with neural networks being less frequently explored compared to classical approaches, as illustrated in Fig. 9. Neural networks, Sparse Spectrum Gaussian Processes, and Linear Regression were applied to predict coronary perfusion pressure in different studies. Among these, neural networks demonstrated superior performance, showcasing their potential, through experiments conducted with variations in datasets and experimental environments. Similarly, for blood pressure estimation tasks, the neural network-based LSTM model showed substantial improvement over traditional methods, highlighting its effectiveness in time-series data handling. Interestingly, RF has been utilized to classify blood flow within specific ranges, treating it as a classification task.

In another experiment, a modified 1D network architecture outperformed SVM, 1D-LeNet5 (in accuracy), and 1D-AlexNet (in computational efficiency) (Zhao et al. 2021). Additionally, an unsupervised approach leveraging the KNN algorithm was used to identify optimal parameter sets based on distance metrics (Jalali et al. 2014). This highlights KNN’s utility in addressing problems with unlabeled data. For supervised classification tasks, CNN and RF were applied in different approaches, with CNN delivering superior performance across various datasets and conditions. On the contrary, for regression tasks, methods such as LSTM, Linear Regression, Neural Networks, and Sparse Spectrum Gaussian Processes were explored, with LSTM achieving the most significant performance gains due to its ability to capture temporal dependencies. Figure 9 illustrates the application of these predictive regression and classification methods. On the other hand, no significant real-life implementation of these approaches has been found, according to our research. However, this analysis underscores the potential of neural network-based models and provides a foundation for future research, offering valuable insights for solving CPR-related problems effectively.

### Pulse and return of spontaneous circulation detection (ROSC)

In the context of CPR, Pulse detection is a vital aspect of CPR, allowing healthcare providers to decide whether to continue chest compressions or transition to alternative interventions. This involves checking for a palpable pulse to assess blood flow and cardiac activity within 10 s to minimize interruptions. However, detecting weak or irregular pulses can be challenging, especially in high-stress scenarios or low-perfusion patients (Wei et al. 2017). Return of Spontaneous Circulation (ROSC) marks the successful restoration of effective cardiac activity and blood flow after cardiac arrest. It is identified through indicators like a palpable pulse, spontaneous breathing, improved skin color, or increased end-tidal CO2 (EtCO2) levels, often measured using capnography, and may be confirmed with blood pressure or ultrasound (Sell et al. 2010). Pulse detection is directly linked to ROSC recognition, as it helps determine whether circulation has been restored or chest compressions should continue. ROSC, a critical milestone in CPR, signals the heart’s return to an organized rhythm and effective function. ML can improve both pulse detection and ROSC recognition by analyzing real-time physiological data, such as ECG signals and EtCO2 trends, identifying patterns humans may miss (Isasi et al. 2021a). These interconnected processes benefit from ML’s ability to provide accurate, automated assessments, enabling faster, data-driven decisions during CPR and improving patient outcomes.

Table 9 noted the contributing ML techniques for these tasks’ execution. While, regarding ML techniques, ML class, key contribution, and Metrics have been discussed for each of the approaches. For instance, Isasi et al. (2021b) and Sashidhar et al. (2021a) utilize ML techniques to identify a pulse seamlessly during CPR, aiming to avoid disruptions in the process. Their approaches aim to ensure continuous and effective resuscitation efforts. In pursuit of minimizing interruptions in the CPR process, Alonso et al. (2020) introduce a reliable classifier for prompt pulse/no-pulse decisions, with the potential to enhance survival rates. Recently, there has been a notable introduction of the concept of pseudo-PEA, characterized by organized electrical activity accompanied by mechanical activity that is insufficient to produce a detectable pulse (Elola et al. 2019). Elola et al. (2020) conducted a subsequent investigation, developing a RF algorithm to distinguish between ROSC, Pulseless Electrical Activity (PEA), and pseudo-PEA. Additionally, these advancements highlight the potential of ML in analyzing intricate signals and extracting crucial information, thereby improving decision-making in emergency cardiac care. In another context, (Shao et al. 2022) is among the scarce studies employing Reinforcement Learning (RL) in CPR. They introduce a deep RL model grounded on a Deep Q-Network (DQN) policy to intelligently diagnose and recommend treatment for cardiac arrest instances, even in incomplete or missing patient information scenarios. Their model aims to optimize CPR success rates and sustain ideal blood pressure levels during pulse detection and ROSC.

#### Dataset

Data for pulse detection and ROSC tasks were primarily collected from hospital patients over extended periods with the support of governing and medical authorities. As a result, most of the experimental datasets remain confidential. However, access to these datasets can often be arranged by contacting the authors of the relevant research papers. Additionally, a publicly available dataset for pulse prediction tasks includes data from 383 patients treated for OHCA (Sashidhar et al. 2021b). This dataset is accessible at^1^Despite the variation in experimental data, certain types, such as ECG and thoracic impedance (TI) signals, are frequently used, as demonstrated in the approaches by (Isasi et al. 2021b), (Sashidhar et al. 2021b), and (Elola et al. 2019). Furthermore, (Elola et al. 2019) also incorporated EtCO2 data as a time-series signal alongside ECG and TI signals. Interestingly, the experiments by (Shao et al. 2022) employed reinforcement learning techniques, leveraging different states before and after CPR as experimental inputs. These datasets were gathered in diverse organizational settings, reflecting variations in availability, facilities, and experimental requirements. Table 10 provides a detailed overview of the datasets, including their organization, data types, and sources.

#### Analytical discussion

Pulse detection and ROSC recognition in CPR are classification tasks in ML, where sensitivity and specificity are critical metrics alongside accuracy. These metrics play a pivotal role in influencing clinical decision-making and patient outcomes. Sensitivity ensures the accurate detection of weak pulses or ROSC, minimizing the risk of missed cases that could delay life-saving interventions. Specificity, on the other hand, reduces false positives, preventing the premature cessation of chest compressions or inappropriate transitions to post-cardiac arrest care. Achieving a balance between these metrics is essential for optimizing CPR efficacy and ensuring timely, accurate responses (Reynolds et al. 2022).

**Table 9:** Comparative Overview of ML approaches for Pulse and ROSC Tasks of CPR

References	AI/ML techniques	ML class	Key contribution	Metrics
Alonso et al. (2020)	SVM	Supervised Learning	Pulse detection	Sen, Spe, BAC, Acc
Sashidhar et al. (2021b)	Linear discriminant analysis-LDA	Supervised Learning	Predicting pulse status	AUCs, Sen, Spe
Elola et al. (2019)	RF	Supervised Learning	Pulse detection	AUC, Sen, Spe
Isasi et al. (2021b)	RF	Supervised Learning	Pulse detection	Sen, Spe, BAC
Shao et al. (2022)	DQN	RL	Improving cardiac emergency care	Mean reward, Median reward

**Table 10:** Highlights about the datasets used for non-invasive blood pressure and chest compression

References	Data type	Data source	Data organization
Alonso et al. (2020)	ECG and TI Signals	Hospital patients (Human)	Amplitude Resolution per least significant bit-Sampling and sampling frequency of ECG and TI signals, 1.03mV and 250 Hz, and 0.74m and 200 Hz, respectively
Sashidhar et al. (2021b)	ECG and TI Signals	Patients (Human)	ECG Pulse check after 10 s and 5 s; sampling rates ranging from 125 to 250 Hz
Elola et al. (2019)	ECG, TI and the EtCO2 Signals	Human	ECG, TI and capnography sampling frequencies of 250 Hz/200 Hz & 125 Hz respectively
Isasi et al. (2021b)	ECG and TI Signals	Hospital patients	ECG and TI resampled to 250 Hz; CPR interval at 3 s & 12.5s
Shao et al. (2022)	States and values during IHCA, CPR, ROSC, and ROSC to hospitalization, hospitalization to discharge, and follow-up after discharge	The Utstein mode of IHCA and CPR registration forms	Data divided into two stages: Stage 1, time was set to 60 min with time step 2 min, and Stage 2, time was set to 24 h with time step 30 min

**Fig. 10:**
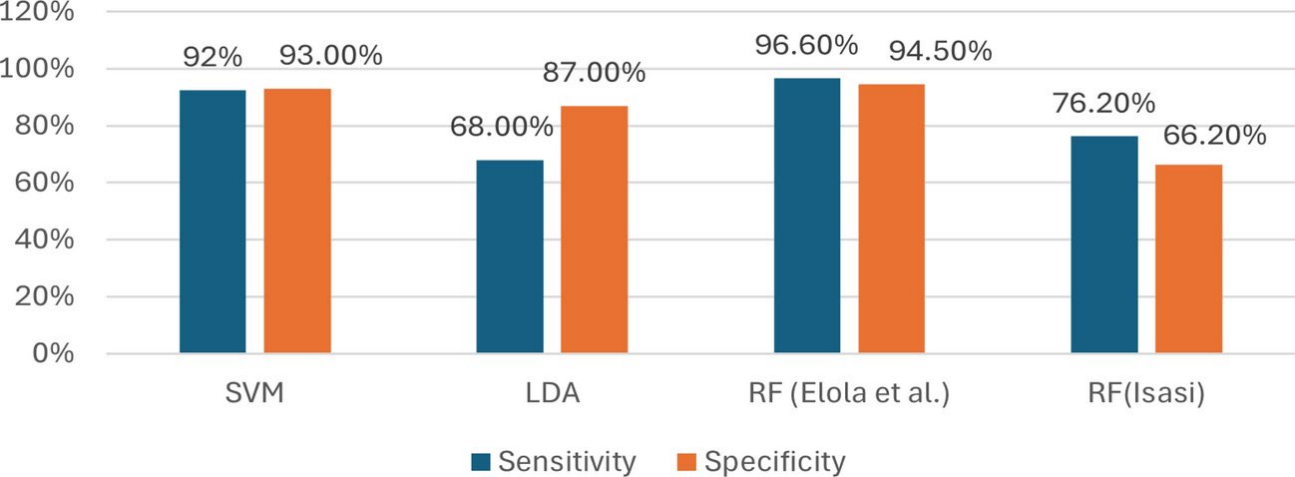
Analysis of model performance based on Sensitivity and specificity score for the pulse detection & ROSC tasks

Figure 10 illustrates the sensitivity and specificity scores for various models and approaches in pulse detection and ROSC tasks. Among these, the RF algorithm from Elola et al. (2019) demonstrated the highest balance between sensitivity and specificity. However, the same algorithm, as applied by Isasi et al. (2021b), exhibited the lowest scores, though the data and experimental environment were different in both cases. It highlights the significant impact of variations in implementation, approaches, data, and experimental environments on performance. Additionally, the SVM approach from Alonso et al. (2020) achieved over 90% in both metrics, indicating a strong balance. Conversely, the Linear Discriminant Analysis (LDA) model by Sashidhar et al. (2021b) showed a notable gap between sensitivity and specificity, suggesting limitations in its application under specific conditions. These findings underscore the influence of dataset characteristics, experimental environments, and algorithmic implementation on model performance. However, our research found no significant real-world implementation of these approaches. The analysis will provide valuable insights for selecting suitable approaches and algorithms for similar tasks in future applications, emphasizing the need to carefully evaluate trade-offs between sensitivity and specificity in ML-driven CPR applications

#### ML approaches for other tasks in CPR

In addition to the previously discussed applications, ML has been employed for various other CPR-related tasks. These include early detection of cardiac arrest, assessing the need for CPR based on emergency call analysis, and addressing specialized considerations such as pediatric CPR care. Table 11 shows and explores several other ML approaches for other CPR tasks. For instance, Blomberg et al. (2019), Blomberg et al. (2021), Chin et al. (2021), and Byrsell et al. (2021) investigated the use of ML as a supportive tool to recognize cardiac arrest in emergency calls, highlighting the potential of AI in early intervention using supervised method. Bender et al. (2020) proposed an ML algorithm to improve patient-centric pediatric CPR.

**Table 11:** Comparative overview of ML approaches for other tasks of CPR

References	AI/ML techniques	Key contribution	Task description
Bender et al. (2020)	SVM	Enhance child-focused CPR care	Identify pediatric ventricular fibrillation types
Blomberg et al. (2019)	Automatic speech recognition (ASR) model for speech-to-text and an OHCA detector	Early detection of cardiac arrest	Detection of cardiac arrest during emergency phone calls using audio analysis
Blomberg et al. (2021)	ASR model for speech-to-text and an OHCA detector	Improved dispatcher recognition	Enhancing dispatcher’s ability to recognize cardiac arrest during emergency calls
Byrsell et al. (2021)	ASR model for speech-to-text and an OHCA detector for real-time prediction	Support dispatchers in cardiac arrest recognition	Assisting dispatchers in identifying cardiac arrest from emergency call data
Chin et al. (2021)	SVM	Early recognition of caller’s emotion	Analyzing caller’s emotions during emergency calls to aid in early cardiac arrest detection
Islam et al. (2025)	Autoencoder(multi-modal)	Denoising CPR signals	Simulate CPR signal, Remove artifacts from CPR signals significantly by preserving correlations between signals and improving explainability
Kim et al. (2023)	XGBoost, Random Forest(RF), Stochastic Gradient Boosting(GBM)	Neurological outcomes of survivors after CPR	Using mean blood pressure to classify Poor neurological outcomes in patients who underwent CPR

The studies discussed in this section underscore the potential of ML and AI in revolutionizing CPR. From early detection to intervention and post-event analysis, these technologies pave the way for more personalized and effective resuscitation strategies.

## Future prospects, challenges and discussion

After thoroughly exploring current ML methods for improving CPR, we have looked into the prospects of how these approaches can naturally make CPR more effective. We examined various existing ML strategies and identified those paying close attention to supervised learning. However, the availability of labeled data is very difficult considering real-life CPR scenarios. In contrast, there is limited experimentation with unsupervised and self-supervised learning approaches. This challenge arises due to the critical nature of CPR events, where data collection is often urgent and unstructured, making manual annotation complex and time-consuming. Additionally, ethical and privacy concerns limit access to high-quality, labeled datasets for training ML models. Moreover, current CPR research employs traditional ML methods like RF, KNN, SVM, LSTM, and CNN, but lacks exploration of Transformer-based architectures, which might offer superior capabilities in handling sequential and contextual data, potentially revolutionizing resuscitation research. In addition, only one paper has been identified concerning the application of the RL approach for CPR optimization. Despite the potential for the utilization of RL in automating and optimizing CPR procedures has still not been explored significantly. Compared to traditional ML techniques RL offers a dynamic decision-making framework that continuously adapts to patient responses and changing conditions during CPR. Unlike traditional models, which rely on predefined features and labeled datasets, RL can learn optimal policies through trial and error, making it particularly suited for real-time and sequential decision-making. Furthermore, Human-aware AI and explainability are important concerns in the future, especially in considering critical decision-making tasks in medical applications in general (Zaki et al. 2023) and the CPR context using ML in particular. In this section, we will investigate all of these possibilities that RL could offer for CPR, as well as explore other AI techniques such as transformers and Explainable AI (XAI), highlighting their significance in the context of CPR.

### Potentiality of reinforcement learning

**Fig. 11:**
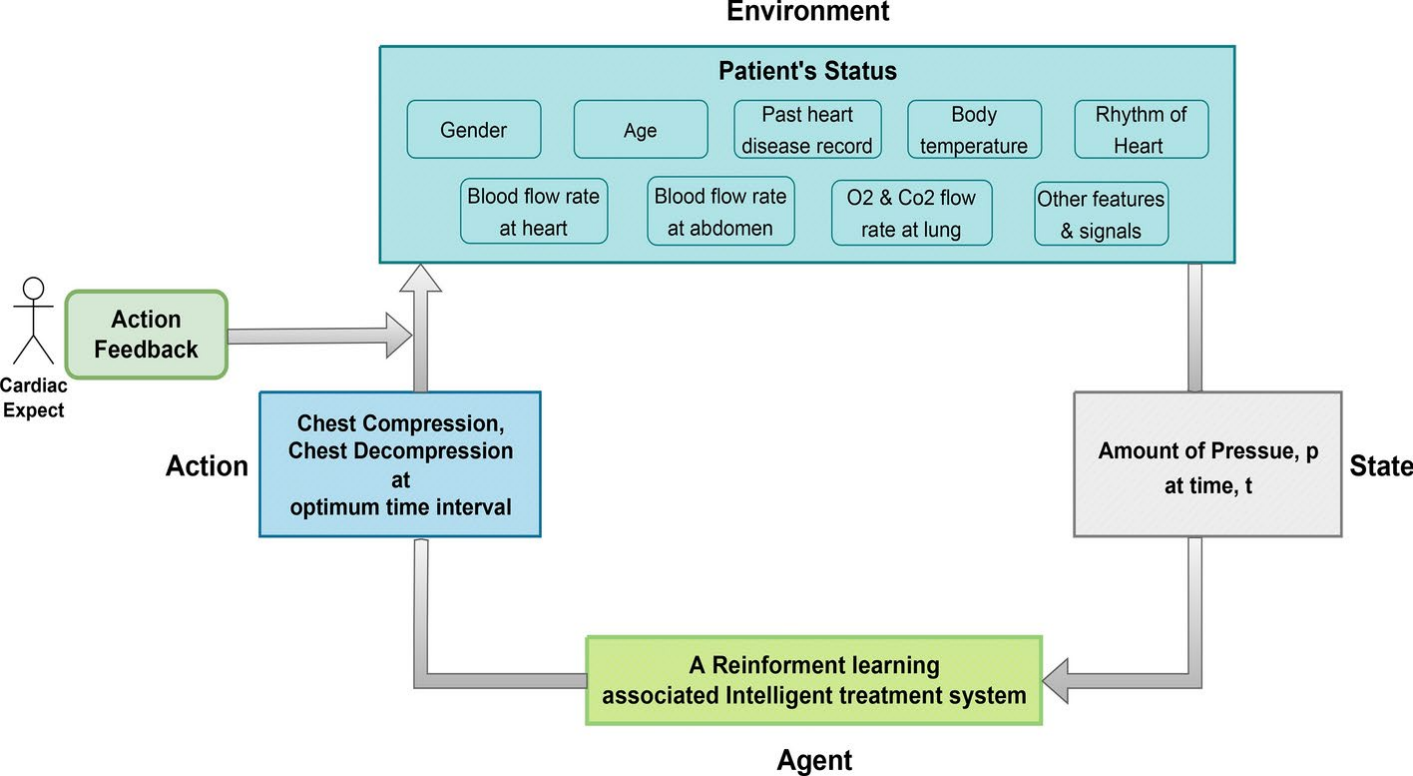
A human feedback reinforcement learning associated CPR optimization system

RL algorithms can be highly effective in optimizing CPR techniques by learning from simulated and real-world CPR scenarios. The RL agent, representing the model, receives feedback on compression depth, rate, and other patient-specific parameters, dynamically adjusting its actions to maximize the likelihood of successful resuscitation. By continuously refining compression force, timing, and response adaptability, RL enhances CPR effectiveness. Moreover, RL can determine the optimal amount of pressure on the chest and abdomen to increase blood flow to the heart, ensuring the most efficient resuscitation technique, where timing intervals play a crucial role. Figure 11 illustrates a human-feedback RL system associated with CPR optimization.

Human Feedback Reinforcement Learning (HFRL) can further enhance CPR by incorporating real-time guidance from human practitioners. In CPR scenarios, HFRL adaptively refines CPR techniques based on direct feedback from healthcare providers, improving the accuracy and effectiveness of CPR administration over time. This approach leverages medical expertise to enhance the learning process of RL algorithms, ensuring that CPR techniques remain optimized and tailored to individual patient needs and response patterns (Griffith et al. 2013; Bai et al. 2022).

Moreover, RL is instrumental in the development of adaptive CPR protocols that dynamically adjust based on patient responses and evolving clinical conditions. By recognizing patterns in physiological data, the RL agent can optimize compression parameters and recommend interventions accordingly. Analyzing large datasets with RL algorithms enables the identification of patterns linked to successful outcomes, aiding in the continuous improvement of CPR quality, protocol refinement, and real-world guideline updates (Wiering and Van Otterlo 2012). Furthermore, RL algorithms possess the capability to analyze patient-specific data and develop personalized resuscitation plans, leading to CPR strategies customized to individual patient characteristics. Multiple RL methods can effectively accomplish these tasks (Li 2017; Arulkumaran et al. 2017).

Recent advancements in RL have further broadened its applicability, demonstrating the potential for real-time adaptive decision-making and resource optimization in dynamic and complex environments analogous to CPR scenarios. For instance, (Zhao et al. 2024) proposed a collaborative computation offloading framework employing the Twin Delayed Deep Deterministic policy gradient (TD3) algorithm within multi-UAV-assisted mobile edge computing (MEC) environments. Their work addresses challenges in computation offloading, resource allocation, and UAV energy management, effectively utilizing RL’s capability to optimize complex real-time decision-making processes under constrained conditions. Such methodologies indicate promising avenues for employing similar RL approaches to CPR, emphasizing the development of advanced adaptive protocols that dynamically optimize CPR administration in real-time scenarios. Exploring these RL-driven solutions represents an important future direction to enhance CPR efficiency and patient outcomes.

#### Policy-based methods

##### Policy gradient methods

Policy Gradient Methods play a crucial role in training adaptive policies that guide healthcare providers during resuscitation efforts. These methods learn from observed states, such as patient vital signs, to directly map to optimal actions, such as adjusting chest compression depth and rate. They are well-suited for continuous and dynamic action spaces, making them effective for improving the quality of chest compressions (Sutton et al. 1999). By shaping reward functions to encourage desirable actions, addressing uncertainty, and accommodating patient-specific conditions, these methods contribute to personalized and effective CPR strategies. Their adaptability, online learning capabilities, and potential for transfer learning across scenarios make Policy Gradient Methods valuable tools for enhancing decision-making in dynamic and critical CPR environments (Peters and Schaal 2006; Gu et al. 2017).

##### Actor-critic methods

These methods integrate both value-based (Q-learning) and policy-based approaches, where the *actor* (policy) learns optimal compression techniques, and the *critic* evaluates their effectiveness. Actor-Critic models can be particularly useful in learning adaptive resuscitation protocols by continuously refining strategies based on real-time physiological feedback (Grondman et al. 2012; Kumar et al. 2020).

#### Value-based methods

##### Policy optimization algorithms and deep deterministic policy gradient (DDPG)

Policy Optimization Algorithms and DDPG are highly effective in learning optimal CPR strategies based on observed patient states. These methods are particularly beneficial for handling *continuous action spaces*, such as dynamically adjusting compression depth and rate. Unlike traditional RL models that rely on discrete actions, DDPG enables *smooth and real-time adjustments* to improve CPR administration (Schulman et al. 2017; Achiam et al. 2017). Both Policy Optimization Algorithms and DDPG support adaptive and personalized CPR strategies by refining reward structures, handling uncertainties, and enabling continuous online learning (Li et al. 2019; Tan 2021).

#### Meta-learning and inverse reinforcement learning (IRL)

##### Meta-reinforcement learning (Meta-RL)

Meta-RL offers the ability to adapt to new CPR scenarios quickly with minimal data. This capability is essential in resuscitation settings where patient conditions and responses vary widely. Meta-RL models can *generalize CPR strategies* across different patient cases, ensuring that resuscitation protocols remain effective even in novel situations (Mitchell et al. 2021).

##### Inverse reinforcement learning (IRL)

IRL helps in developing *expert-informed CPR strategies* by learning from medical practitioners’ demonstrations. By analyzing past successful resuscitations, IRL models can infer optimal actions that *mirror expert decision-making*, ensuring alignment between AI-driven recommendations and human expertise. This approach improves *model interpretability* and allows AI systems to provide more clinically relevant CPR guidance (Zhifei and Joo 2012; Arora and Doshi 2021).

#### Additional applications of RL in CPR

Beyond compression optimization, RL can assist in strategic *AED* placement. By analyzing historical cardiac arrest data, RL models can recommend optimal AED placement locations, improving accessibility and reducing response times (Cummins 1989). Additionally, RL-based approaches can be applied in *emergency dispatch systems*, predicting patient deterioration risks and guiding proactive intervention planning in *real-time emergency scenarios*.

### Transformers and explainable AI

On the other hand, our investigation of ML approaches for future applications identified a significant gap. Although methods like RL, KNN, SVM, LSTM, and CNN have been used in CPR research, experiments with Transformer-based architectures are notably absent. Transformers have shown promise in capturing and understanding sequential and contextual information in the long-range, which can be effective in CPR scenarios. Their strong ability to analyze temporal and dynamic data suggests that incorporating Transformer-based architectures could greatly enhance the range of ML methods used in resuscitation research.

Emerging technologies like Transformers and RL present significant opportunities to address longstanding challenges in CPR.

- Transformers: These architectures, known for their ability to capture sequential and contextual information, can process dynamic physiological data such as ECG and blood pressure signals. Their attention mechanisms enable the prioritization of critical information during CPR interventions.
- Reinforcement learning: RL can optimize decision-making in real-time, adjusting compression depth and rate based on patient-specific data. Adaptive learning through human feedback can further enhance the effectiveness of CPR protocols.

#### Potentiality of transformers

Transformers, designed for the efficient processing of sequential data, hold significant promise in capturing long-range dependencies and temporal patterns crucial for decision-making, which can be vital in CPR signal processing (Vaswani et al. 2017; Lin et al. 2022). Specifically, the attention mechanism in Transformers can prioritize critical segments of CPR sequences, such as sudden changes in coronary perfusion pressure or heart rhythm irregularities, ensuring that the model focuses on the most relevant information during real-time analysis. This capability is particularly beneficial in dynamic scenarios where patient responses to interventions can vary rapidly (Liu et al. 2023). Moreover, pre-training on large biomedical datasets and fine-tuning for CPR-specific tasks allow Transformers to leverage knowledge from diverse healthcare contexts, enabling the detection of subtle patterns in CPR signals that might otherwise be overlooked (Han et al. 2021; Ruan and Jin 2022). For instance, Transformers can integrate multi-modal inputs, such as ECG, blood pressure, and respiratory rate, to create a holistic representation of the patient’s condition, supporting real-time, data-driven decision-making during CPR.

The interpretability of attention weights adds an additional layer of transparency, allowing healthcare professionals to understand and trust the model’s decision-making process. This is particularly valuable in life-saving interventions, where explainability can support clinical judgments. Transformers also facilitate continuous learning, adapting their understanding of evolving patient conditions during resuscitation efforts and providing ongoing support for decision-making. In addition, Transformers’ ability to process data in parallel enables them to deliver quick and accurate real-time predictions, which is crucial in time-sensitive CPR scenarios (Islam et al. 2023). Recent studies have demonstrated the potential of Transformer models in related biomedical tasks, such as ECG classification and respiratory signal analysis (Che et al. 2021; Vrba and Robinson 2001). These studies highlight their effectiveness in improving the accuracy and efficiency of signal interpretation, suggesting that similar benefits can be achieved for CPR data processing. Further exploration and real-world application of Transformer-based models could unlock significant advancements in CPR signal analysis, enabling more precise, context-aware interventions.

#### Relevance of explainable AI

Looking forward into the future, at least two central themes for research linking ML to CPR seem to be of interest to make progress. We have seen how ML algorithms may yield important guidance for administering CPR. But it will be practitioners who actually take actions and make decisions based on what the ML systems indicate. This means that providing effective interfaces for the human users to view what the ML systems propose will be quite important (Nuutinen and Leskelä 2023; Bienefeld et al. 2023). And as practitioners are increasingly asked to adjust their decisions according to the advice of AI systems, a really essential goal will be to support providing explanations of the reasoning of those AI systems (known as XAI or explainable AI) in a way that the human users can comprehend. The more that our AI systems migrate to embracing DL, the more challenging it will be to actually explain the inner workings of those systems (Contreras et al. 2023). If solutions based on Large Language Models (LLMs) really do become more commonplace for any healthcare applications of ML, there are at least some ideas currently for how to generate those explanations (Huang et al. 2023) as a promising starting point. The introduction of attention-based transformer models can highlight the relevance of particular contexts in use but here too some XAI research has explored how to work with these approaches (Chefer et al. 2021; Alammar 2021) so that XAI within CPR should be effective as well. The fact that other researchers have begun to tackle human-AI partnerships in the setting of emergency medicine is also very encouraging (Okada et al. 2023; Määttä et al. 2023). The study of human-aware AI may pose challenges to resolve in full and systems in use that yield more straightforward output are certainly important as our first steps, but the path ahead with more forward-looking AI models is one that we can seek to explore, in order to secure important acceptance of these systems in all their forms, into the future.

The uses of AI systems outlined in this paper are clearly intended to provide support to medical experts, so that these individuals will still be making the final decisions about actions to take. This fits well with the concept of algorithm-based or algorithm-driven decisions (and not what is known as algorithm-determined decisions) per a distinction made by the German Data Ethics Committee (Amann et al. 2022) and reflected on at length in the FDA report of the US (Food and (FDA) 2021). Moreover, future AI-driven CPR applications should align with established frameworks such as the FDA’s regulations on AI in medical devices (Handley et al. 2024), the EU AI Act (Edwards 2021), and IEEE AI ethics standards (Zhang and Zhang 2023) to ensure compliance, patient safety, and ethical responsibility. We anticipate as well a need to continuously revise the solutions for explainable AI to be effective for medical professionals due to the evolving nature of the technology (Amann et al. 2020)). The algorithms themselves can become more personalized by making use of data about the specific patient at hand but this is true of any effective AI system being used for healthcare; one simply has to recognize which of the data concerning a patient is worth merging into the decision-making algorithms that are being designed (Arbelaez Ossa et al. 2022). If there are any concerns with diagnostic performance, then one can consider alternatives (Ploug and Holm 2020).

This deeper exploration of the care required when introducing explanations of AI systems is part of the more general ethical concerns that arise when AI is employed in healthcare. If algorithms rely on historical data of other patients, one wants to avoid issues of bias for instance; this can be an entirely separate consideration with approaches applied to properly understand the data-gathering process (Collins et al. 2021; Norori et al. 2021). Moreover, the approaches that we discuss which have AI methods informing medical experts of the probability of certain events occurring for the patient, when explained, would indicate which factors were being considered and with which readings. Once more, it would up to those experts to make the final medical decisions with this information on hand. As has been suggested when AI is introduced in other medical contexts, some kind of session explaining the technology at hand and its range of possible uses is desirable so that there is a true partnership between medical and AI experts once the technology is put into place; educating medical students about AI is also helpful in this respect (Alowais et al. 2023).

### Discussion and conclusion

In future CPR research, a major obstacle is the difficulty of obtaining real-life CPR data. Accessing high-quality, labeled data for CPR scenarios is challenging due to privacy regulations and ethical considerations surrounding medical data. In addition, it is difficult to get large amounts of data classifying CPR procedures into successful and unsuccessful administrations. This scarcity of large and diverse datasets that accurately represent various CPR situations makes it hard to train robust ML models. To overcome these challenges, generative models such as VAEs can help create large amounts of data points resembling the existing small amount of real-life data. Moreover, synthetic data generation techniques can be delicate along with real-life data, whereas more mathematical models such as Babbs provide representations of CPR scenarios, allowing the generation of realistic synthetic data (Babbs et al. 1984; Fitzgerald et al. 1981). Additionally, data augmentation techniques offer a promising strategy to enhance datasets, enabling extensive research in CPR. The scarcity of data is not the only challenge; selecting the right data is also complicated. The use of Self-Directed Machine Learning (SDML) is one of the ML solutions that may be adopted in the future in CPR applications, as it supports medical staff in selecting proper data and making the right decisions in a secure and robust manner (Zhu et al. 2022).

There has been significant progress in cardiac rhythm analysis during CPR using ML/DL techniques, particularly in determining shock/no-shock decisions based on cardiac rhythm. Recent studies have primarily focused on cleaning and restoring ECG signals to improve rhythm classification, as CPR-induced artifacts can significantly degrade signal quality. However, a key limitation is the availability of high-quality, real-world data. Many studies use generated or augmented data, which may not fully capture the complexity of actual CPR artifacts. Furthermore, the available datasets often include a limited number of subjects, which restricts model generalization. Larger, more diverse datasets, incorporating various CPR artifacts, and data from multiple AED devices, are essential for improving performance and ensuring robustness across different clinical settings. Additionally, DL models, while effective, come with a high computational cost due to their complexity and the need for extensive hyperparameter tuning, making them challenging to deploy in real-time or resource-constrained environments.

To conclude, this paper highlights the potential of integrating ML with life-saving CPR techniques during cardiac arrest. By thoroughly reviewing and analyzing existing ML applications in CPR, we have identified key research gaps and unexplored opportunities that could pave the way for significant advancements in the biomedical field. The unique contribution of this survey compared to previous literature lies in its structured and comprehensive interdisciplinary synthesis of advanced ML methodologies explicitly applied to CPR. Unlike prior surveys that typically focus broadly or solely on conventional resuscitation methods, our review categorizes and critically evaluates recent innovations, emerging research trends, and real-world implementation challenges, offering clear guidance for future research directions. Our comprehensive synthesis underscores the importance of interdisciplinary collaboration between computer science and medical research and sets a strategic framework for future studies. We believe that the continued exploration and implementation of ML in CPR will improve resuscitation outcomes, enhance the efficiency and effectiveness of emergency medical interventions, and ultimately save more lives.

## Footnotes

1 https://zenodo.org/
